# Genetic Resources in the “Calabaza Pipiana” Squash (*Cucurbita argyrosperma*) in Mexico: Genetic Diversity, Genetic Differentiation and Distribution Models

**DOI:** 10.3389/fpls.2018.00400

**Published:** 2018-03-29

**Authors:** Guillermo Sánchez-de la Vega, Gabriela Castellanos-Morales, Niza Gámez, Helena S. Hernández-Rosales, Alejandra Vázquez-Lobo, Erika Aguirre-Planter, Juan P. Jaramillo-Correa, Salvador Montes-Hernández, Rafael Lira-Saade, Luis E. Eguiarte

**Affiliations:** ^1^Departamento de Ecología Evolutiva, Instituto de Ecología, Universidad Nacional Autónoma de México, Mexico, Mexico; ^2^Unidad de Biotecnología y Prototipos, Facultad de Estudios Superiores Iztacala, Universidad Nacional Autónoma de México, Mexico, Mexico; ^3^Departamento de Conservación de la Biodiversidad, El Colegio de la Frontera Sur, Villahermosa, Mexico; ^4^Centro de Investigación en Biodiversidad y Conservación, Universidad Autónoma del Estado de Morelos, Cuernavaca, Mexico; ^5^Campo Experimental Bajío, Instituto Nacional de Investigaciones Forestales, Agrícolas y Pecuarias, Celaya, Mexico

**Keywords:** *Cucurbita*, cultivated squash, genetic diversity, genetic structure, nuclear microsatellites, species distribution models

## Abstract

Analyses of genetic variation allow understanding the origin, diversification and genetic resources of cultivated plants. Domesticated taxa and their wild relatives are ideal systems for studying genetic processes of plant domestication and their joint is important to evaluate the distribution of their genetic resources. Such is the case of the domesticated subspecies *C. argyrosperma* ssp. *argyrosperma*, known in Mexico as *calabaza pipiana*, and its wild relative *C. argyrosperma* ssp. *sororia*. The main aim of this study was to use molecular data (microsatellites) to assess the levels of genetic variation and genetic differentiation within and among populations of domesticated *argyrosperma* across its distribution in Mexico in comparison to its wild relative, *sororia*, and to identify environmental suitability in previously proposed centers of domestication. We analyzed nine unlinked nuclear microsatellite loci to assess levels of diversity and distribution of genetic variation within and among populations in 440 individuals from 19 populations of cultivated landraces of *argyrosperma* and from six wild populations of *sororia*, in order to conduct a first systematic analysis of their genetic resources. We also used species distribution models (SDMs) for *sororia* to identify changes in this wild subspecies’ distribution from the Holocene (∼6,000 years ago) to the present, and to assess the presence of suitable environmental conditions in previously proposed domestication sites. Genetic variation was similar among subspecies (*H*_E_ = 0.428 in *sororia*, and *H*_E_ = 0.410 in *argyrosperma*). Nine *argyrosperma* populations showed significant levels of inbreeding. Both subspecies are well differentiated, and genetic differentiation (*F*_ST_) among populations within each subspecies ranged from 0.152 to 0.652. Within *argyrosperma* we found three genetic groups (Northern Mexico, Yucatan Peninsula, including Michoacan and Veracruz, and Pacific coast plus Durango). We detected low levels of gene flow among populations at a regional scale (<0.01), except for the Yucatan Peninsula, and the northern portion of the Pacific Coast. Our analyses suggested that the Isthmus of Tehuantepec is an effective barrier isolating southern populations. Our SDM results indicate that environmental characteristics in the Balsas-Jalisco region, a potential center of domestication, were suitable for the presence of *sororia* during the Holocene.

## Introduction

Domestication is an ideal model to study evolution because it is usually fast and gradual (Purugganan and Fuller, 2011; Meyer and Purugganan, 2013; Gaut, 2015; Gaut et al., 2015). Population genetics studies allow to analyze the dynamics of the domestication process and to make inferences about the origins and histories of crops (Meyer and Purugganan, 2013; Aguirre-Liguori et al., 2016).

Sometimes the ancestral wild populations can still be studied along with the domesticated forms and varieties, allowing paired comparisons between populations under different selection processes in the same environment (Aguirre-Liguori et al., 2016). Also, the coexistence and possibility of hybridization of domesticated taxa and their wild relatives allows having a source of genetic variation during domestication, increasing genetic diversity and the presence of alleles of agronomic value (Warschefsky et al., 2014). Nevertheless, the possibility of hybridization raises questions, such as: (1) How do domesticated and wild relatives remain genetically differentiated? and (2) How frequent is introgression among wild and domesticated relatives? It is also important to mention that signals of domestication may be confused by long-distance human-mediated dispersal and by intermittent crosses between domesticated and wild taxa, sometimes making it difficult to disentangle the history of domestication (Besnard et al., 2013; Meyer and Purugganan, 2013). As domestication and crop improvement involve genetic bottlenecks (Gaut et al., 2015), they can lead to a reduction of genetic diversity and increased inbreeding. During domestication, crops are transported from their center of domestication to new environments, which may lead to new local adaptation that in some cases can be achieved through introgression with their wild relatives or other domesticated relatives (Gaut et al., 2015).

The mechanisms for the maintenance of the genetic differentiation among domesticated populations and their wild relatives have seldom been studied. It has been proposed that in some species, such as *Cucurbita argyrosperma* and *Zea mays*, gene flow is asymmetric, being more frequent from the wild to the domesticated taxa (Montes, 2002; Hufford et al., 2013). Moreover, Hufford et al. (2013) found resistance to gene flow from domesticated maize into wild teosinte, which could be explained by low gene flow rates, by the fact that domesticated genes are not advantageous for wild taxa, or strong selection by humans against hybrids. Cruz-Reyes et al. (2015) observed that domesticated-wild hybrids of *Cucurbita* showed lower reproductive output. Hufford et al. (2013) found that many alleles that characterize domesticated varieties are found at lower frequencies in their wild relatives, suggesting that the attributes associated with domestication are not produced by *de novo* mutations, but constitute part of the standing genetic variation of wild taxa (Doebley et al., 2006).

Surveys of genetic variation of wild populations and their cultivated relatives is a first step for the description of genetic resources, such as analyzing how much genetic variation is still found in domesticated taxa compared to their wild relatives, their degree of differentiation, and evaluating how much ancestral and ongoing gene flow (hybridization) exists among wild and domesticated taxa (Warschefsky et al., 2014). These topics are relevant for the management of domesticated populations and for the future preservation of genetic resources (Warschefsky et al., 2014; Govindaraj et al., 2015). Moreover, these comparative studies are the first step toward understanding the origin and diversification of domesticated plant taxa. Current molecular tools, along with population genetics and modern phylogeographic approaches, allow understanding the distribution of genetic variation from an evolutionary perspective (Eguiarte et al., 2013; Aguirre-Dugua and González-Rodríguez, 2016; Aguirre-Liguori et al., 2016).

The study of crop origins has traditionally involved identifying geographic areas of high diversity and sampling populations of wild progenitor species (Kraft et al., 2014). Linking genes, crops, and landscapes through a geographical analysis of genetic data is one important way to achieve multilevel integration (Van Etten and Hijmans, 2010; Hufford et al., 2012; Besnard et al., 2013). Furthermore, species distribution modeling, projected into past conditions, offers a view of the potential geographic pattern of taxa during the domestication process (Hufford et al., 2012; Kraft et al., 2014).

The genus *Cucurbita* (pumpkins, squashes, and gourds), with 20 taxa of perennial or annual plants, is native to the Americas. Mexico is considered its center of origin and diversification (Lira-Saade, 1995; Mapes and Basurto, 2016). *Cucurbita* represents an interesting system for the study of domestication (Lira et al., 2016b) with five different domesticated species: *C. pepo*, *C. moschata*, *C. ficifolia*, *C. maxima*, and *C. argyrosperma* (Wilson et al., 1992; Sanjur et al., 2002; Kocyan et al., 2007; Gong et al., 2013; Zheng et al., 2013; Kates et al., 2017). Cucurbits were some of the first plants domesticated in the Americas, ca. 10,000 years ago (Smith, 1997; Zizumbo-Villarreal et al., 2016). Within the genus *Cucurbita*, each domestication event occurred independently, sometimes on more than one occasion (Lira et al., 2016b). Today, domesticated cucurbits still have a fundamental role in the diet of people in Mexico, Central and South America, and in many other regions of the world, and they are considered an essential phytogenetic resource (FAO, 2010).

**FIGURE 1:**
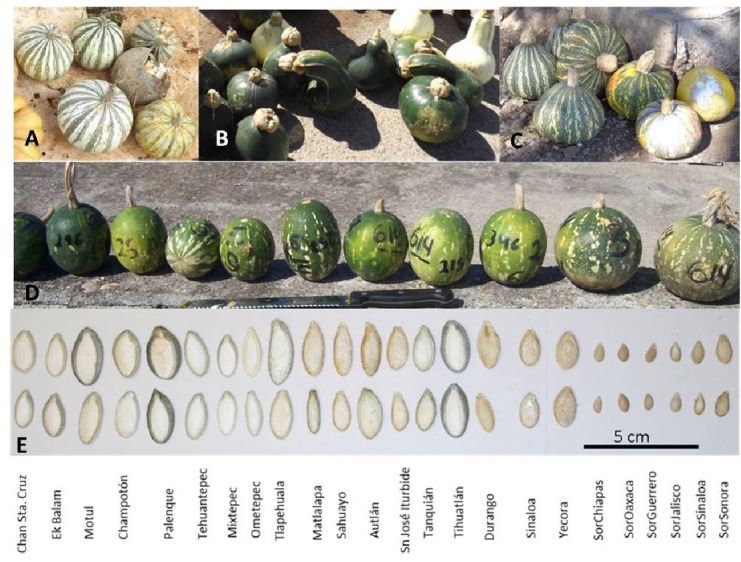
Morphological characteristics of subspecies *C. argyrosperma* ssp. *argyrosperma* from Yucatán **(A)**, Sonora **(B)** and Jalisco **(C)**, and *C. argyrosperma* ssp. *sororia* **(D)** from Jalisco. Seeds from each population are shown at the bottom **(E)**. Population ID is shown in **Table 1**.

**FIGURE 2:**
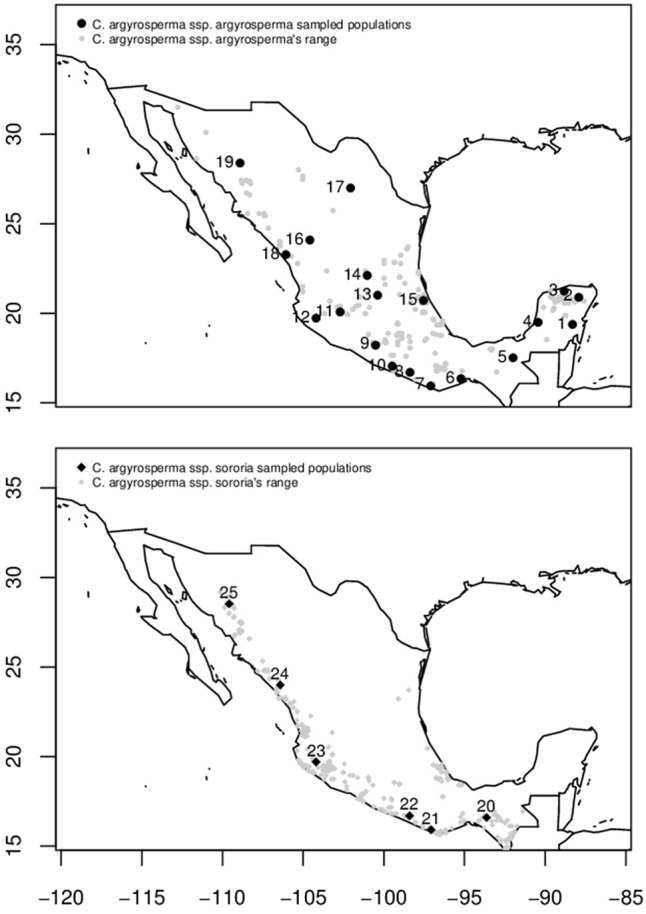
Distribution area in Mexico (gray symbols) and sampled populations of *C. argyrosperma* ssp. *argyrosperma* (**Top**, black points) and *C. argyrosperma* ssp. *sororia* (**Bottom,** black diamonds). Population ID is shown in **Table 1**.

Among domesticated cucurbits, *C. argyrosperma*, known in Mexico as *calabaza pipiana* or *calabaza mixta*, is highly appreciated for its seeds, which are used in Mexican gastronomy. Also, fruits are medicinal, commercial, and food resources (Lira-Saade, 1995; Villanueva, 2007). It is a species with cultural and economic importance both locally and worldwide. The oldest evidence of domestication for this species is ∼8,600 years old from the Xihuatoxtla shelter, in the state of Guerrero (Rannere et al., 2009). This is a highly diverse species in form, color and size of its seeds and fruits (Lira-Saade, 1995; **Figure 1**). *C. argyrosperma* is currently divided into two subspecies: the domesticated *C. argyrosperma* ssp. *argyrosperma* (*argyrosperma* hereafter) and its wild relative *C. argyrosperma* ssp. *sororia* (*sororia* hereafter; **Figure 1**) (Organization for Economic Co-operation and Development [OECD], 2012; Gong et al., 2013; Zheng et al., 2013; Kates et al., 2017). Both wild and cultivated subspecies can be found in tropical and semi desert regions from the Southeastern United States through Mexico and northern Central America, reaching Nicaragua, from sea level to 1,700 m above sea level (Villanueva, 2007; Lira et al., 2016b). These subspecies have a sympatric distribution in most of their range, except for the Yucatan peninsula, where the wild subspecies is absent (Lira-Saade, 1995; Organization for Economic Co-operation and Development [OECD], 2012; **Figure 2**).

*Cucurbita argyrosperma* is an important crop in local agriculture systems in Mexico and in other countries in the Americas. It is grown and selected in traditional ways. It is commonly found as a seasonal crop, but irrigation is used in some areas. A large amount of its production is not reported because it is used in subsistence agriculture in Mexico and Central and South America (Montes, 1991, 2002; Villanueva, 2007; Organization for Economic Co-operation and Development [OECD], 2012). In other regions of the world it is not extensively cultivated because of the low quality of its flesh (Lira-Saade, 1995; Organization for Economic Co-operation and Development [OECD], 2012), but there are records of some genetically improved cultivars grown in the United States and Canada. Some improved lines show differences in fruit and seed size, shape, and color, such as “Green Striped Cushaw,” “White Cushaw,” “Magdalena Striped,” “Papago,” “Japanese Pie,” “Hopi,” “Taos,” “Parral Cushaw,” “Veracruz Pepita,” and “Silver Seed Gourd” (Organization for Economic Co-operation and Development [OECD], 2012).

Nevertheless, there are few studies focused on analyzing the genetic resources of cucurbits and covering most of their distributions (Bellon et al., 2009; Lira et al., 2016b). Only a few studies have analyzed the genetic variation of *C. argyrosperma*, including an analysis at a local scale (a region in the state of Jalisco) using isozymes (Montes-Hernández and Eguiarte, 2002), which found that *argyrosperma* has less genetic variation (*H*_E_ = 0.35–0.41) than its wild relative (*H*_E_ = 0.433), and low levels of genetic differentiation among populations (*F*_ST_ = 0.077). Two studies, one based on isozymes in commercial cultivars (Decker-Walters et al., 1990), and another with accessions using RAPDs (Cerón et al., 2010), found lower genetic diversity in *argyrosperma* (*H* = 0.039 and 0.063 for isozymes and RAPDs, respectively) than in other domesticated taxa of the genus, such as *C. moschata* (*H* = 0.052 and 0.11 for isozymes and RAPDs, respectively) and in *C. pepo* (*H* = 0.068 and 0.104 for isozymes and RAPDs, respectively) (Decker-Walters et al., 1990; Cerón et al., 2010). Recently, Balvino-Olvera et al. (2017) studied wild populations of *sororia* along the Pacific coast of Mexico with microsatellites, reporting high levels of genetic variation (*H*_E_ = 0.756) and higher genetic diversity and heterogeneity among southern populations in the states of Guerrero and Oaxaca. Clearly, there is a lack of population-based genetic diversity analyses that include both the cultivated and wild *C. argyrosperma* throughout its range.

The main aim of this study was to use molecular data (microsatellites) to assess the levels of genetic variation within and among populations of domesticated *argyrosperma* across its distribution in Mexico. We also analyzed populations of its wild relative, *sororia*, to compare levels of genetic variation and differentiation. Additionally, we estimated the levels of recent gene flow among populations and subspecies, and performed projections of the wild subspecies’ distribution area in the mid-Holocene (∼6,000 years ago), in order to identify environmental suitability in previously proposed domestication centers, such as the Balsas-Jalisco region based on archeological records (Sanjur et al., 2002; Piperno et al., 2009; Rannere et al., 2009). We expected to find lower genetic diversity and higher levels of inbreeding in cultivated *argyrosperma* than in its wild relative *sororia*, in accordance with a previous study (Montes-Hernández and Eguiarte, 2002). In addition, we expected to find lower genetic differentiation among populations in the same geographic area than in more distant areas, and signals of on-going gene flow among subspecies, as reported by Montes (2002) and Montes-Hernández and Eguiarte (2002).

## Materials and Methods

### Sampling and DNA Extraction

We obtained seeds from at least 3 fruits (one fruit from each different plant) from 19 populations of cultivated landraces of *argyrosperma* and 6 wild populations of *sororia* representative of the species distribution in Mexico (**Figure 2** and Supplementary Table S1). Ten populations were obtained from the germplasm collection of the Instituto Nacional de Investigaciones Forestales, Agrícolas y Pecuarias (INIFAP), Campo Experimental Bajío, in 2014 (BG in Supplementary Table S1). Fruits from 15 additional populations were collected in the field between 2013 and 2015. Sampled wild populations were located close to cultivars of *argyrosperma* to assess levels of gene flow among subspecies. All the collected fruits were stored in greenhouse conditions at the Institute of Ecology, UNAM, until they became ripe. Between 5 and 20 seeds from each collected fruit were grown in commercial substrate under greenhouse conditions (35°C in average) for 40 days, and young leaves were collected for DNA extraction. DNA extraction was performed using a modified CTAB protocol (Doyle and Doyle, 1987). For nuclear microsatellite loci, we genotyped a total of 440 individuals, 327 of which were attributed to *argyrosperma* and 113 to *sororia*.

### Microsatellite Analyses

We amplified 12 of the nuclear microsatellite loci reported by Gong et al. (2008) for *C. pepo* (Supplementary Table S2). Loci were selected from different chromosomes to improve genome coverage and to reduce the probability of linkage disequilibrium. For better results, we selected only highly variable dinucleotide loci. We used a multiplex approach for microsatellite amplification in a 15 μl final volume, consisting of 1× Buffer, 1.2 mM MgCl_2_, 0.2 mM of dNTPs, 0.13 μM of each primer (six primers per multiplex, forward primers were marked with one of the following fluorescent dyes: 6-FAM, HEX and VIC), 1 μl of Taq polymerase (PROMEGA) and 10 ng of genomic DNA. Amplification reactions were performed in a Veriti 96-well Thermal cycler (Applied Biosystems) with the following program: 95°C for 5min, followed by 35 cycles of 95°C for 40s, 60°C (Ta) for 40s, 75°C for 55s, and a final step of 72°C for 5min followed by 4°C. To control for possible contamination, we used blank controls for each reaction. All products were verified in 2 % agarose gels and PCR products were sent to the Roy J. Carver Biotechnology Center at the University of Illinois, United States for genotyping^1^. Electropherograms were analyzed with PeakScanner (Applied Biosystems) to build a matrix with the genotypes of each individual.

### Null Alleles and Measures of Genetic Diversity

We conducted a null allele analysis using the method proposed by Chakraborty et al. (1992) implemented in the Microchecker v2.2.3 (Van Oosterhout et al., 2004). In addition, we performed a Hardy-Weinberg exact test and a linkage disequilibrium test using Arlequin v. 3.0 (Excoffier et al., 2005). We obtained allele frequencies by direct estimation using Arlequin v. 3.0, and determined the number of private alleles for each population and subspecies by direct count from the allele frequencies data. We also obtained descriptive statistics, such as the proportion of polymorphic loci per population (*P*), allelic richness (*A*), and the expected (*H*_E_) and observed (*H*_O_) heterozygosities with the same software, and estimated the inbreeding coefficient (*F*_IS_) for each population using Genepop 4.0 (Rousset, 2008). In addition, we obtained the rarefied allelic richness with ADZE 1.0 (Szpiech et al., 2008) accounting for the lowest population size of six individuals.

### Genetic Differentiation and Genetic Structure

To assess the genetic structure among subspecies and among populations we used Structure v 2.3.4 (Pritchard et al., 2000). This program uses Bayesian probability to assign individuals to different genetic clusters (*K*) based on allele frequencies without considering the population of origin. We performed previous runs to assess the best combination of priors to be used for the analysis and the length of the Markov Chain Monte Carlo (MCMC) chains. Accordingly, we performed a final run with admixture and correlated allele frequencies as priors, and without considering the putative population of origin of each individual. We used a burn-in of 500,000 chains and 1,000,000 MCMC chains, and tested values of *K* from 1 to 10, and 10 repetitions for each *K*. The results were run through Structure Harvester v 0.6.93 (Earl and vonHoldt, 2012), and the results from the Evanno test (Evanno et al., 2005) were considered to determine the value of *K* that showed fit to our data. We performed an analysis of molecular variance (AMOVA; Excoffier et al., 1992) considering the genetic clusters obtained with Structure.

As an additional test to identify the number of genetic groups formed by our data, we used the adegenet library to perform discriminant analysis of principal components (DAPC; Jombart et al., 2010). DAPC is a multivariate analysis that summarizes the genetic differentiation between groups. This analysis identifies genetically related individuals by partitioning the within group and among group genetic variation (Jombart et al., 2010). We performed two independent DAPC analyses. The first analysis included all individuals to assess the relationship among subspecies. We conducted a cross-validation test to determine the number of PCs to be retained. Accordingly, we retained 40 PCs and two discriminant functions. For the second analysis, we excluded the populations that showed high genetic differentiation to allow depicting the relationship among populations within *argyrosperma*. We retained 25 PCs and two discriminant functions in accordance to the cross-validation test.

We estimated the genetic differentiation among populations through pairwise *F*_ST_ using adegenet (Jombart, 2008; Jombart and Ahmed, 2011) for R v.1.4.2 (R Core Team, 2016). To depict the genetic relationships among populations, we used the pairwise *F*_ST_ matrix to construct a dendrogram with the complete agglomeration method using the *hclust* function in the package ape (Paradis et al., 2004) for R. To determine the degree of statistical support for internal nodes we made an UPGMA dendrogram with R v.3.2.0, and evaluated 1000 trees constructed from bootstrap resampling of the loci with this same library.

To test for isolation by distance in each subspecies, we used ade4 (Dray and Dufour, 2007) for R to perform a Mantel test with 999 permutations. For this test, we first used the Geographic Distance Matrix Generator (Ersts, 2011) to transform sample coordinates into a geographic distance matrix. We also performed an AMOVA (Excoffier et al., 1992) testing different scenarios to determine whether subspecies, or genetic clusters provide a better explanation of the genetic variance in the species *C. argyrosperma*.

### Gene Flow

To obtain estimates of the migration rates among populations and subspecies, we used BayesAss v.3.0.4 (Wilson and Rannala, 2003). This program, based on Bayesian probability, detects immigrant ancestors up to two generations in the past, even if overall genetic differentiation is low. An advantage of this approximation is that it does not assume that populations have reached equilibrium (Rannala and Mountain, 1997), which may be the case for species that have undergone rapid demographic expansion, such as domesticated taxa. We performed several runs to determine the best number of MCMC, to tune the priors and to check for convergence. Accordingly, we performed 30,000,000 MCMC iterations, with a burn-in of 3,000,000 and a sampling frequency of 2,000. We set the parameters as follows: deltaA = 0.70 (mixing parameter for allele frequencies), deltaF = 0.90 (mixing parameter for inbreeding coefficient), deltam = 0.05 (mixing parameter for migration rates) to obtain an acceptance rate between 0.2 and 0.6, as suggested by Rannala (2007). We obtained a trace file to check for convergence with Tracer v.1.5^2^ (Rambaut and Drummond, 2009).

To detect barriers to gene flow, we used the Monmonier algorithm (Monmonier, 1973; Manni et al., 2004) implemented in adegenet for R, considering both subspecies and for each subspecies separately. The Monmonier algorithm conducts a heuristic search used to define barriers based on dissimilarity scores. First, the genetic distance between contiguous populations is computed and the two populations with the highest level of differentiation are used to specify the starting boundary of the barrier. Then, the barrier is followed to both ends until either end reaches the edge or a barrier. These steps are repeated until the within-group sum of squares indicates that regional subdivision has progressed considerably (Monmonier, 1973). We used the *optimize.monmonier* function, which uses different starting points to find the solution that better explains genetic distances among populations based on the largest sum of local distances. We used values of pairwise *F*_ST_ as the distance matrix to perform the analysis, and the number of starting points set to 10 for *argyrosperma* and to three for *sororia* (i.e., half of the number of populations).

### Species Distribution Models

To assess environmental suitability in possible areas of domestication we used species distribution models (SDMs) projections into the mid-Holocene (6,000 years ago) for the wild relative *sororia*. We constructed a database with geographic coordinates of collected and known *sororia* populations. Points from Central America were downloaded from GBIF^3^, and 699 points from Mexico were obtained from Salvador Montes-Hernández, for a total of 720 occurrence. This database was purged to eliminate duplicated pixels. In addition, to ensure that all points fell within the species distribution, we estimated Mahalanobis distances using previously selected environmental variables (see below for selection methodology). The points deviating by two standard deviations or more from the mean were mapped, checked, and discarded if they fell outside the species range. We finally retained 273 occurrence points to perform the SDMs (Supplementary Figure S1).

To reduce the uncertainty associated with SDMs, it is necessary to select only the more informative and uncorrelated climatic variables (Hirzel et al., 2002). To do so, we downloaded the set of 19 bioclimatic variables taken from the worldwide temperature and rainfall data within the WorldClim 1.4 dataset (Hijmans et al., 2005). To determine which climatic variables to use, we analyzed the 273 occurrence points in a principal component analysis (PCA) and a Spearman correlation matrix. For the PCA, we considered as informative the components that, taken together, represented 87% of the variance associated with the data. For the Spearman correlation matrix, we defined an uncorrelated model by using a threshold of *r* < 0.85 (Booth et al., 1994). Nine bioclimatic layers were selected: Mean Temperature of Warmest Quarter, Mean Temperature of Coldest Quarter, Isothermality (BIO2/BIO7) (^∗^100), Maximum Temperature of Warmest Month, Precipitation of Wettest Month, Precipitation of Driest Month, Precipitation Seasonality, Precipitation of Warmest Quarter, and Precipitation of Coldest Quarter.

We generated SDMs for current and past climate conditions with MaxEnt 3.3.3 (Phillips et al., 2004, 2006), using the 273 occurrence points and nine bioclimatic variables. We limited the analysis by cropping all climate layers to the distribution of *sororia* (9.51003°N to 34.18357°N and -116.1351°W to -70.80147°W). MaxEnt was executed using a 20% random test rate, 30 replicates, replicated bootstraps, 1000 maximum iterations and a convergence threshold of 0.00001, with extrapolation and clamping turned off. The distribution model was derived from the average model and evaluated using the score of the area under the curve (AUC; Elith et al., 2006).

For SDM projecting to mid-Holocene climate conditions, we downloaded the layers corresponding to atmospheric-ocean general circulation models (AOGCM) based on the Community Climate System Model CCSM4 (Collins et al., 2006), which incorporates dynamics of atmospheric processes, including radiation, convection, condensation and evaporation. This AOGCM has already been used in the reconstruction of past distributional models in the region (Waltari et al., 2007; Peterson and Nyári, 2008; Waltari and Guralnick, 2009; Holmgren et al., 2010; Gámez et al., 2014; Scheinvar et al., 2017). All environmental analyses were performed at a resolution of 30 arcsec (∼1 km^2^).

In order to create a presence/absence map, we used the 95th percentile value of observed sample points as a threshold for the logistic model. This value assumes that up to 5% of the records used for generating the model are subject to error. For current and mid-Holocene times, we generated presence/absence maps for *sororia.* To identify areas with suitable environmental conditions for the species under current and past climate conditions, we performed a sum of the SDMs, thus highlighting the areas with potential stability conditions from the mid-Holocene to the present.

## Results

### Genetic Diversity

Three microsatellites (CMTp175, CMTm187, and CMTm144) showed evidence of null alleles and were therefore excluded from further analyses. As expected, there was no evidence of linkage disequilibrium among loci.

All loci showed significant deviations from Hardy-Weinberg equilibrium (HWE) in at least one population (Supplementary Table S3). Nevertheless, we performed multiple comparisons, which show that loci with significant deviations from HWE are different among populations.

We obtained a total of 84 alleles for the nine analyzed loci. At least one locus was monomorphic in each population (**Table 1**). We found higher levels of polymorphism per population (*p* = 0.02) in the cultivated populations of *argyrosperma* (*P* = 0.775 ± 0.39 SD; **Table 1**) than in the wild *sororia* (*P* = 0.641 ± 0.11 SD; **Table 1**). For *argyrosperma*, the proportion of polymorphic loci ranged between 0.44 and 0.88. For *sororia*, the proportion of polymorphic loci ranged between 0.55 and 0.77.

**Table 1:** Genetic diversity obtained for 9 nuclear microsatellite loci for *C. argyrosperma* ssp. *argyrosperma* and *C. argyrosperma* ssp. *sororia.*

State	Population	Population ID	*n*	*P*	*A*	*RA*	*Pa*	*H*_O_	*H*_E_	*F*_IS_
Quintana Roo	Chan_Sta_Cruz	(1) Chan	21	0.44	4.0 (2.1)	1.6 (0.76)	2	0.318 (0.21)	0.524 (0.19)	0.39^∗^
Yucatán	Ek_Balam	(2) Ek	22	0.77	2.1 (0.37)	1.4 (0.16)	0	0.311 (0.29)	0.250 (0.16)	-0.253^∗^
Yucatán	Motul	(3) Mot	22	0.88	2.75 (0.70)	1.6 (0.28)	0	0.278 (0.21)	0.303 (0.20)	0.08
Campeche	Champoton	(4) Champ	22	0.77	3 (2.2)	1.7 (0.78)	1	0.246 (0.19)	0.342 (0.25)	0.29^∗^
Chiapas	Palenque	(5) Pal	20	0.88	2.62 (0.73)	1.8 (0.34)	2	0.437 (0.29)	0.369 (0.21)	-0.18^∗^
Oaxaca	Tehuantepec	(6) Teh	20	0.88	3.3 (1.9)	1.8 (0.27)	4	0.316 (0.17)	0.348 (0.16)	0.08
Oaxaca	Mixtepec	(7) Mix	21	0.77	2.4 (0.535)	1.6 (0.23)	1	0.292 (0.22)	0.331 (0.18)	0.11
Guerrero	Ometepec	(8) Ome	17	0.88	2.6 (1)	1.8 (0.32)	0	0.525 (0.31)	0.420 (0.18)	-0.26^∗^
Guerrero	Tlapehuala	(9) Tla	10	0.77	2.8 (0.9)	1.8 (0.66)	0	0.428 (0.33)	0.462 (0.23)	0.078
Guerrero	Matlalapa	(10) Mtp	18	0.88	2.5 (0.75)	1.8 (0.28)	0	0.494 (0.27)	0.422 (0.17)	-0.173^∗^
Michoacán	Sahuayo	(11) Sah	22	0.66	2.6 (0.81)	1.6 (0.31)	0	0.454 (0.25)	0.374 (0.18)	-0.22^∗^
Jalisco	Autlán	(12) Aut	21	0.66	4.3 (2.8)	1.9 (1.1)	2	0.365 (0.24)	0.449 (0.28)	0.19^∗^
Guanajuato	San José Iturbide	(13) SJI	6	0.66	2.1 (0.40)	1.6 (0.22)	0	0.472 (0.28)	0.482 (0.12)	0.022
San Luis Potosí	Tanquian	(14) Tan	22	0.77	2.7 (1.4)	1.7 (0.49)	0	0.285 (0.17)	0.356 (0.18)	0.2^∗^
Veracruz	Tihuatlán	(15) Tih	23	0.88	3.27 (1.0)	1.71 (0.16)	5	0.228 (0.16)	0.315 (0.11)	0.28^∗^
Durango	Durango	(16) Dgo	6	0.88	2.1 (0.35)	1.36 (0.05)	1	0.395 (0.26)	0.464 (0.13)	0.15^∗^
Coahuila	Cuatrocienegas	(17) CCC	22	0.88	2.7 (0.88)	NA	3	0.315 (0.31)	0.458 (0.17)	0.31^∗^
Sinaloa	Sinaloa	(18) SinalP	6	0.66	2.1 (0.40)	1.6 (0.76)	0	0.388 (0.32)	0.588 (0.10)	0.39^∗^
Sonora	Yecora	(19) Yec	6	0.77	3 (1)	1.4 (0.16)	2	0.380 (0.27)	0.547 (0.2)	0.32^∗^
***C. argyrosperma* ssp. *argyrosperma***			**327**	**0.775 (0.39)**	**2.786 (0.76)**	**1.6 (0.28)**	**23**	**0.364 (0.25)**	**0.410 (0.16)**	**0.033 (0.06)**
Chiapas	Sor_Chis	(20) SChis	20	0.55	2.8 (0.83)	1.7 (0.78)	1	0.39 (0.28)	0.425 (0.10)	0.084
Oaxaca	Mixtepec	(21) Soax	21	0.55	3.4 (1.4)	1.8 (0.34)	3	0.352 (0.32)	0.502 (0.14)	0.303^∗^
Guerrero	Ometepec	(22) Sgro	22	0.77	2.8 (1.8)	1.8 (0.27)	0	0.233 (0.25)	0.300 (0.21)	0.225^∗^
Jalisco	Autlán	(23) Sjal	22	0.66	3 (1.2)	1.6 (0.23)	1	0.431 (0.28)	0.447 (0.16)	0.035
Sinaloa	Culiacán	(24) SoSin	13	0.77	3.2 (1.3)	1.8 (0.32)	2	0.527 (0.35)	0.501 (0.21)	-0.053
Sonora	Alamos	(25) SoSon	15	0.55	2.4 (0.5)	1.8 (0.66)	1	0.400 (0.16)	0.394 (0.13)	-0.013
***C. argyrosperma* ssp. *sororia***			**113**	**0.641 (0.11)**	**2.93 (1.17)**	**1.8 (0.28**)	**8**	**0.388 (0.27)**	**0.428 (0.15)**	**0.077 (0.16)^∗^**


**The table shows the State and Population ID of collected samples, number of individuals (n), proportion of polymorphic loci (P), allelic richness (A), corrected allelic richness (RA), Number of private alleles (Pa), observed heterozygosity (H_*O*_), expected heterozygosity (H_*E*_), and inbreeding coefficient (F_*IS*_), (^∗^) significant at p < 0.05. Standard deviation (SD) is shown in parenthesis. NA could not be estimated by the program due to the presence of missing data.**

Forty alleles were private to *argyrosperma* and eleven were found only in *sororia*. Mean number of private alleles per population was 1.21 in *argyrosperma* and 1.33 in *sororia*. Within *argyrosperma*, populations Tih and Teh (full name of geographic locations are shown in **Table 1**) showed the highest number of private alleles. Within *sororia*, populations Soax and SoSin showed the highest number of private alleles. Overall, the populations from Oaxaca showed the highest proportion of private alleles in both subspecies. Allelic richness was similar (*p* = 0.23) in cultivated *argyrosperma* (*A* = 2.786 ± 0.76 SD) and in wild *sororia* (*A* = 2.93 ± 1.17 SD). For *argyrosperma*, rarefied allelic richness ranged from 1.9 in Aut to 1.36 in Dgo (**Table 1**). For *sororia*, rarefied allelic richness ranged from 1.8 in four populations to 1.6 in Aut (**Table 1**).

Mean observed and expected heterozygosity were similar among subspecies (*H*_O_ = 0.388 and *H*_E_ = 0.428 in *sororia*, and *H*_O_ = 0.36 and *H*_E_ = 0.410 in *argyrosperma*; *H*_O_ *p* = 0.484; *H*_E_ *p* = 0.656). For *argyrosperma*, genetic diversity (*H*_E_) ranged from 0.588 in SinalP to 0.25 in Ek. For *sororia*, mean genetic diversity (*H*_E_) was 0.428, with the highest value in Soax (0.502) and the lowest in Sgro (0.3) (**Table 1**).

Both subspecies showed similar overall inbreeding coefficients (*F*_IS_ = 0.033 ± 0.069 SD, *p* = 0.34 in *argyrosperma* and *F*_IS_ = 0.077 ± 0.16 SD, *p* = 0.33 in *sororia*, and were not statistically different *p* = 0.656). Within *argyrosperma*, five populations exhibited heterozygosity excess, while nine showed heterozygote deficiency and the rest were in HWE (**Table 1**). The highest values for heterozygosity deficiency were found in Chan and SinalP (*F*_IS_ = 0.39) and Yec (*F*_IS_ = 0.32) and for heterozygosity excess the lowest values were found in Ek and Ome (*F*_IS_ = -0.253 and -0.26, respectively; **Table 1**). Within *sororia*, two populations exhibited heterozygosity deficiency, while the rest were in HWE (**Table 1**). The highest *F*_IS_ value was found in Soax (0.303) (**Table 1**).

### Population Structure

**FIGURE 3:**
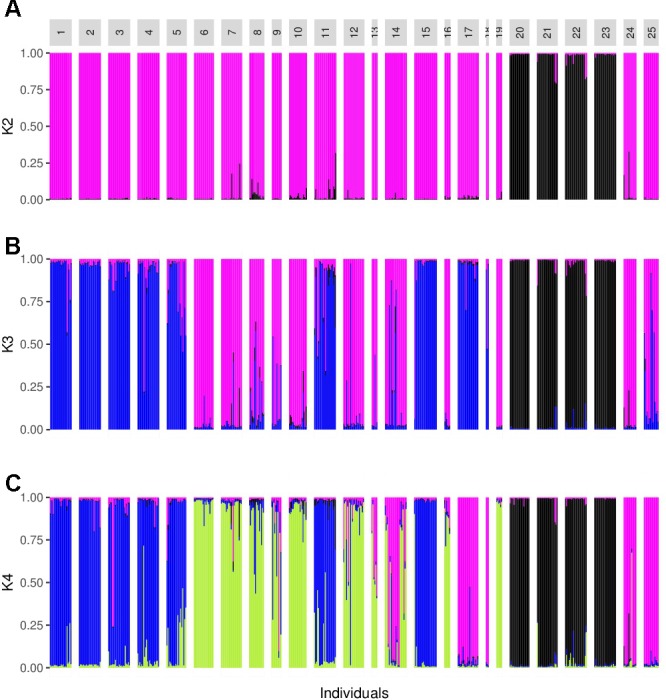
Barplot from the Structure analysis. **(A–C)** *K* = 2, *K* = 3, and *K* = 4. Population ID are as shown in **Table 1**.

**FIGURE 4:**
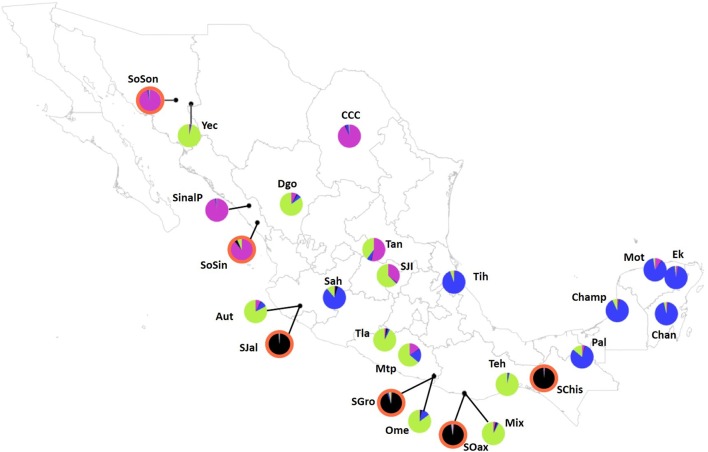
Population map depicting pie graphs corresponding to the proportion of individuals per population assigned to each genetic cluster obtained with STRUCTURE for *K* = 4. Populations with graph pie outlined in orange highlight *sororia* populations. The black cluster consists of four *sororia* populations: Schis, Soax, Sgro, Sjal. The other two *sororia* populations (SoSin and SoSon) were assigned to a second cluster with *argyrosperma* populations CCC, and SinalP located in Northern Mexico (pink). The third cluster (blue) consists of populations Ek, Mot, Chan, Champ and Pal, from the Yucatan Peninsula and populations from Michoacán (Sah) and Veracruz (Tih). The fourth cluster (green) is constituted by populations Teh, Mix, Ome, Tla, Aut, and Yec, from the Pacific coast and Durango (Dgo). Population ID are as shown in **Table 1**.

The analysis performed with Structure suggested a value of *K* = 2, followed by *K* = 4 (**Figure 3**). For *K* = 2 there was a clear genetic differentiation between subspecies (**Figure 3A**), except for the *sororia* populations SoSin and SoSon, that were more similar to *argyrosperma*. The Structure barplot for *K* = 3 shows that within *argyrosperma*, the populations from the Yucatan peninsula, Chiapas, Veracruz, Michoacán and CCC constituted a cluster, while the populations from northern and central Mexico formed another cluster (**Figure 3B**). Finally, for *K* = 4 the clusters largely corresponded to subspecies’ geographic distributions (**Figures 3**, **4**). The first cluster consisted of four *sororia* populations: Schis, Soax, Sgro, Sjal (black in **Figure 4**). The other two *sororia* populations (SoSin and SoSon) were assigned to a second cluster with *argyrosperma* populations CCC, and SinalP located in northern Mexico (pink in **Figure 4**). The third cluster consisted of *argyrosperma* populations from Ek, Mot, Chan, Champ and Pal, from the Yucatan Peninsula and populations from Michoacán (Sah) and Veracruz (Tih) (blue in **Figure 4**). The fourth cluster was constituted by populations Teh, Mix, Ome, Tla, Aut, and Yec, from the Pacific coast and Durango (green in **Figure 4**). The results from this analysis showed some degree of admixture among populations, particularly within *argyrosperma* (populations Tan, SJI and Mtp in **Figure 3C**), but the populations from *sororia* had low levels of admixture with the domesticated subspecies.

**FIGURE 5:**
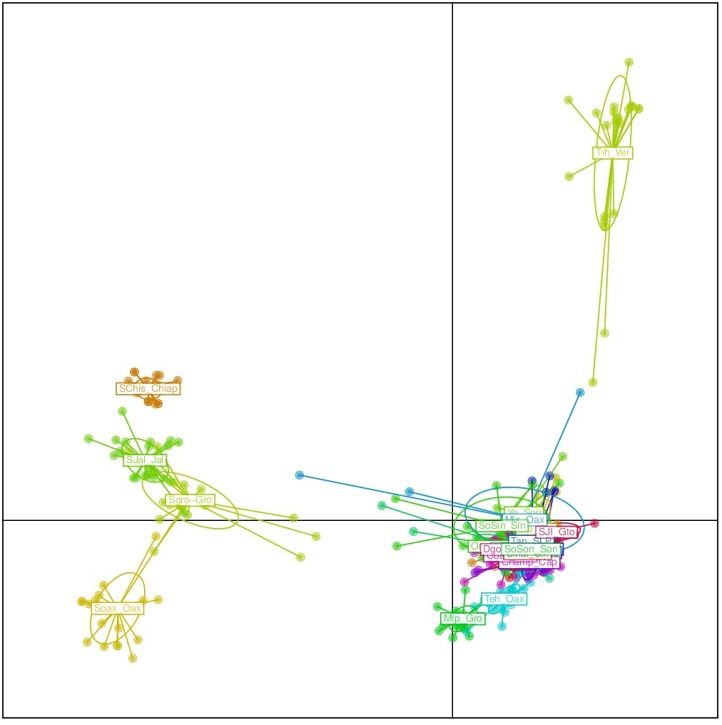
Discriminant analysis of principal components (DAPC) for 19 *argyrosperma* and 6 *sororia* populations. Names starting with S or So correspond to *sororia* populations. Four *sororia* populations are well differentiated from the *argyrosperma* populations.

**FIGURE 6:**
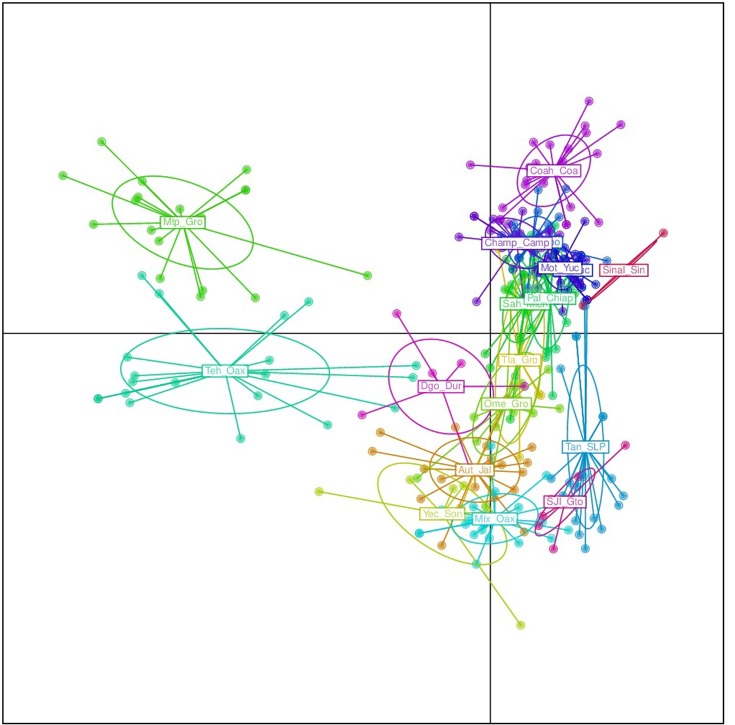
Discriminant analysis of principal components (DAPC) for only 18 *argyrosperma* populations (excluding Tih). Mtp and Teh from the states of Guerrero and Oaxaca, respectively, are well differentiated. Population ID are as shown in **Table 1**.

The results from the DAPC analysis were consistent with the results from Structure (**Figure 5**). Four *sororia* populations were clearly differentiated from *argyrosperma*, while two populations clustered within *argyrosperma*. All *argyrosperma* populations were grouped together, except for Tih from the state of Veracruz, which seemed in this analysis to be well differentiated from the other populations (**Figure 5**). In this DAPC 97.9% of the variance is explained by 40 PCs. A DAPC analysis considering only *argyrosperma* populations, except Tih (**Figure 6**), showed that Mtp and Tehuantepec from the states of Guerrero and Oaxaca, respectively, were well differentiated. Some populations formed very cohesive clusters, i.e., populations from the Yucatan Peninsula and populations from the Pacific Coast. In this second DAPC 94.6% of the variance is explained by 25 PCs.

**FIGURE 7:**
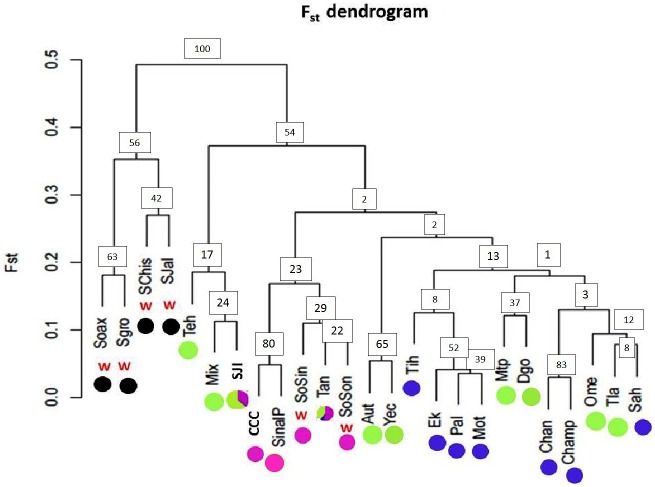
Pairwise *F*_ST_ dendrogram for 19 *argyrosperma* populations and 6 *sororia* populations. Populations of the wild subspecies (*sororia*) are indicated (w). Dot colors agree with the pie graphs corresponding to the proportion of individuals per population assigned to each genetic cluster obtained with STRUCTURE for *K* = 4 shown in **Figure 6**. Population ID is shown in **Table 1**. Node values represent bootstrap support.

Overall genetic structure was higher for wild *sororia* (*F*_ST_ = 0.492, *R*_ST_ = 0.610) than for domesticated *argyrosperma* (*F*_ST_ = 0.264, *R*_ST_ = 0.4). Genetic differentiation among populations (pairwise *F*_ST_) of *argyrosperma* was moderate to high (*F*_ST_ = 0.031–0.515), while genetic differentiation among populations of *sororia* was higher in general (*F*_ST_ = 0.171–0.639). Genetic differentiation among populations of the different subspecies was medium to high (*F*_ST_ = 0.152–0.652; **Figure 7** and Supplementary Table S5). When we estimated genetic differentiation among populations of wild *sororia* without escaped populations (SoSin and SoSon) values were moderate (*F*_ST_ = 0.181–0.352).

The dendrogram built using pairwise *F*_ST_ values (**Figure 7**) showed two well-defined groups: one group including only *sororia* populations from different states along the Pacific coast in Mexico (Oaxaca, Guerrero, Chiapas and Jalisco), and another group of *argyrosperma* populations, including the two *sororia* populations (SoSin and SoSon) mentioned above. Bootstrap values are in general low, as is usually found in intraspecific studies (due to both gene flow and recent common ancestry). Low bootstrap values within *argyrosperma* could also be due to homoplasy and care should be taken with their interpretation. Nevertheless, the two groups had higher support values (above 50%) (**Figure 7**).

An AMOVA that considered each subspecies, explained 20.05% of the genetic variance between subspecies, and most of the variance was found within individuals (50.3%), followed by among populations within subspecies (26.2%), and only 3% of the variance was found among individuals within populations. Given that two putatively wild populations (SoSin and SoSon) were identified as belonging to *argyrosperma* in all genetic structure analyses, and the seeds show an intermediate morphology for size and color (**Figure 1**), we also performed an AMOVA analysis considering these populations as *argyrosperma*. This analysis showed that a higher percentage (30.3%) of the genetic variance was allocated between subspecies; most variance was still found within individuals (46.1%), and less among populations within subspecies (21.4%), finally, 2.3% of the genetic variance is found among individuals within populations. On the other hand, an AMOVA analysis considering the partition suggested by the Structure analysis, *K* = 4, explained 24.5% of the genetic variance among clusters, the variance among populations within clusters was 30.8%, and most of the variance was found within populations (44.6%).

**FIGURE 8:**
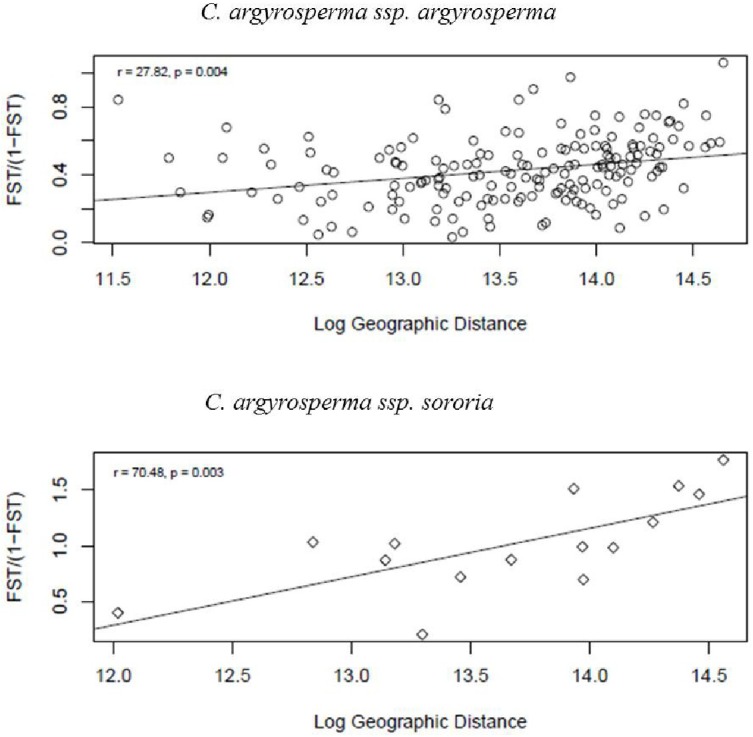
Test of isolation by distance (IBD) obtained through a Mantel test. Results were significant, showing a strong positive correlation between geographic and genetic distances for both *argyrosperma* (*r* = 27.8, *p* = 0.004), and *sororia* (*r* = 70.04, *p* = 0.003).

Mantel tests were significant for both subspecies, indicating spatial structure due to isolation by distance. We found that geographically closer populations are genetically more similar than expected by chance (**Figure 8**).

### Estimates of Gene Flow

Estimates of recent gene flow suggest that the total proportion of migrants for each population was from 17 to 33% (Supplementary Table S4). Nevertheless, in general the proportion of migrants among pairs of populations was low ≤ 0.01; the exceptions were between some populations in the Yucatan Peninsula and in Chiapas, the Pacific coast, and in the northern portion of the Pacific coast, for *argyrosperma*; and in the southern-central portion of the Pacific coast for *sororia* (Supplementary Table S4). The only case where gene flow between cultivated *argyrosperma* and wild *sororia* populations was detected involved the two Northern *sororia* populations (SoSin and SoSon) and a Northern population in San Luis Potosi state, Tan; other analyses strongly suggest that SoSin and SoSon are *argyrosperma* populations escaped from cultivation. This suggests that gene flow between cultivated and truly wild populations is low.

**FIGURE 9:**
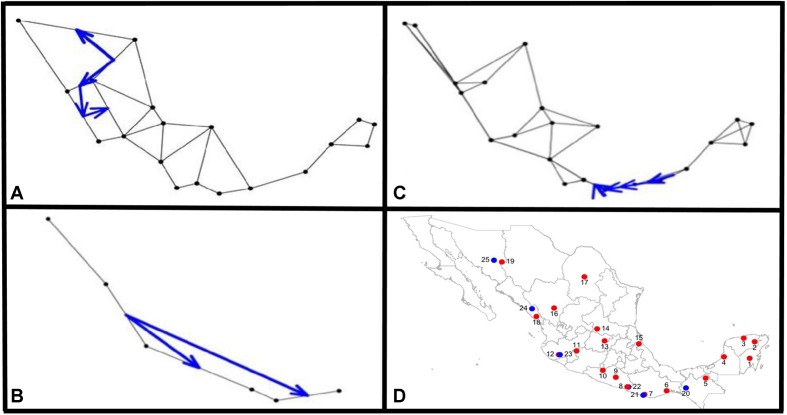
Results from the Monmonier analysis performed with adegenet Black dots represent sampled populations and blue arrows depict significant barriers to gene flow, for **(A)** *C. argyrosperma* ssp. *argyrosperma*, **(B)** *C. argyrosperma* ssp. *sororia*, **(C)** both subspecies. **(D)** Location of populations on a map, blue dots corresponding to *C. argyrosperma* ssp. *sororia* and red dots are *C. argyrosperma ssp. argyrosperma*. Population ID is shown in **Table 1**.

The Monmonier analysis indicated that for *argyrosperma* (**Figures 9A,D**), the northern part of the Sierra Madre Occidental may function as an effective barrier to gene flow, isolating the SinalP and Yec populations. This contrasts with results from the DAPC and structure analyses, where these populations do not seem to be isolated. This can be due to differences in the methodologies, in which Monmonier analysis takes spatial distances into account. For *sororia*, the southern portion of the Sierra Madre Occidental also functions as an effective barrier to gene flow, and isolates population Sjal (**Figure 9B**). When both subspecies were analyzed together, we observed that the main barrier is located in the region of the Isthmus of Tehuantepec, isolating the populations from the Yucatan Peninsula (**Figure 9C**).

### Species Distribution Models

**FIGURE 10:**
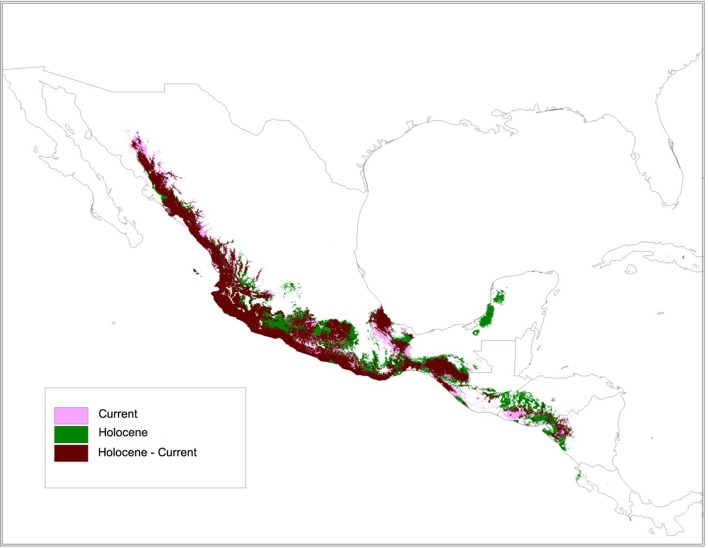
The species distribution model (SDM) for ssp. *sororia* showed stability and good support (AUC: 0.96). Persistence during the mid-Holocene (∼6000 years ago) and present time in light persistence only in the Holocene in purple; and persistence only in the present in green.

The SDM for the wild subspecies, *sororia* (**Figure 10**), showed stability and good support (AUC: 0.96). The SDM projection to the mid-Holocene (∼6000 years ago) suggests that the distribution area of *sororia* has been more or less stable since domestication (**Figure 10**). Nevertheless, the analysis also suggests that *sororia* may have been present in the Yucatan Peninsula during the mid-Holocene and its distribution in the regions of Oaxaca and Guerrero, where the most ancient archeological remains have been found, may have been more continuous than today (**Figure 10**). In addition, the distribution of *sororia* in Central America may have been wider and more continuous from Guatemala and Honduras to the northern area of Nicaragua.

## Discussion

The present study represents the first wide range analysis of the genetic variation, genetic structure and gene flow of *C. argyrosperma*, covering the cultivated *argyrosperma* distribution in Mexico, and including populations of the wild *sororia* distribution in the Pacific Coast from Northern Mexico (Sonora) to Southern Mexico (Chiapas). Our analyses show similar levels of genetic variation in the cultivated populations and in its wild ancestors. Genetic differentiation is higher in wild *sororia* (*F*_ST_ = 0.492) than in domesticated *argyrosperma* (*F*_ST_ = 0.264), but this estimate is probably the product of including two escaped populations (SoSin and SoSon) that were misclassified and analyzed as *sororia.* When we remove these populations, differentiation in wild *sororia* (*F*_ST_ = 0.243) became even lower than the differentiation found in cultivated *argyrosperma.* Gene flow at a regional level is associated to movement of pollen by *Cucurbita* pollinators and to human cultural practices, such as seed exchange among populations (Montes-Hernández et al., 2005; Organization for Economic Co-operation and Development [OECD], 2012). Some patterns of gene flow detected in *argyrosperma* may be the result of these seed exchanges, but these hypotheses should be tested with ethnobotanical data in future analyses.

### Genetic Variation and Inbreeding

Priori et al. (2013) reported an 85% transferability for microsatellites designed for *C. pepo* to cultivated *C. argyrosperma.* Accordingly, nine of twelve microsatellite loci used in this study were adequate for *C. argyrosperma*, while we discarded the additional three because of a high number of null alleles.

Cultivated species often show low levels of genetic variation (Gaut et al., 2015). Surprisingly, both subspecies showed similar levels of genetic diversity (**Table 1**). Comparable values of polymorphic loci, allelic richness and genetic diversity among *argyrosperma* and *sororia* suggests that the subspecies have had similar effective population sizes, and that the theoretical bottleneck associated with domestication was either mild and/or of short duration, followed by a rapid population expansion (Hedrick, 2011).

Genetic variation was similar to what has been reported for other annual plants in microsatellite studies (*H*_E_ = 0.46; Nybom, 2004), but lower when compared to other outcrossing species (*H*_E_ = 0.65; Nybom, 2004). Also, the mean allele number in *C. argyrosperma* was lower than those reported for *C. pepo* using nuclear microsatellite loci (*A* = 3.2–5.6; Formisano et al., 2012; Gong et al., 2012, 2013; Priori et al., 2013; Ntuli et al., 2015) and lower than those reported by Balvino-Olvera et al. (2017) in wild *sororia* populations along the West coast of Mexico (*A* = 12.3). Low levels of allelic richness and genetic diversity in *sororia* may suggest that this species has undergone one or several bottlenecks due to ecological shifts during the Pleistocene, followed by rapid population expansion, as suggested by Kistler et al. (2016). Nevertheless, these comparisons should be taken with caution because analyses were performed with different sets of microsatellite loci, and these hypotheses should be investigated in future studies.

Certain aspects of agricultural management, such as seed exchange, may also affect the levels of genetic variation in *C. argyrosperma* (Montes-Hernández et al., 2005) and in *C. pepo* (Enríquez Cotton, 2017; Enríquez et al., 2017). In particular, the *milpa* system, which predominates in the central and southern portions of Mexico (Lira et al., 2016a), is a form of polyculture (i.e., growing several *Cucurbita* species in the same area) and seed exchange, that can reduce inbreeding at the local level. Nevertheless, it is advisable to perform similar analyses in other wild and domesticated cucurbits to gain further insight into the amount of genetic variation present in *Cucurbita*.

For *argyrosperma*, populations located in the extremes of its distribution (SinalP in Sinaloa and Chan in Quintana Roo) showed the highest levels of genetic variation, while the populations from the Yucatan Peninsula (except Chan) showed the lowest levels of genetic variation. These results, together with the barriers analysis, suggest that the cultivated populations from the Yucatan Peninsula are isolated genetically (Zizumbo-Villarreal and Colunga-GarcíaMarín, 2010; Moreno-Estrada et al., 2014). Moreover, the wild subspecies, *sororia*, is not distributed in the Yucatan Peninsula, thus affecting the potential gene flow among subspecies in this area. In subspecies *sororia* we did not find a geographic pattern for the distribution of its genetic diversity, and Oaxaca was the population that showed the highest genetic variation.

Cultivated species often show high levels of inbreeding (Gaut et al., 2015). Estimates of inbreeding coefficients (*F*_IS_) in *argyrosperma* were highly variable (**Table 1**), with some populations showing heterozygote deficiency (9 populations), as may be expected in a domesticated species, and other populations showing heterozygote excess (5 populations), as previously reported by Montes-Hernández and Eguiarte (2002), may be related to the type of agriculture and management (Cerón et al., 2010), as well as pollinator availability and home range. Heterozygote deficiency could be the result of the short flight capacity of the bees that pollinate these species (Montes, 2002), or to the fact that in traditional subsistence agriculture only a few fruits are selected to plant the next generation (thus, within a field all individuals are highly related; Montes, 2002), while in northern populations the use of improved inbred genetic lines (Servicio de Información Agroalimentaria y Pesquera [SIAP], 2016) could be the cause of heterozygosity deficiency. Negative *F*_IS_ values found in some cases suggests that seed exchange is frequent at a local level (i.e., among neighbor populations) promoting outbreeding, but at a regional level (i.e., among extremes of the distribution) gene flow is low. It will be important to conduct detailed ethnobotanic studies in different regions of the country, along with genetic analysis, to test the effect of agricultural management on the genetic variation of this crop (Montes-Hernández et al., 2005; Bellon et al., 2009).

### Genetic Structure

When populations are isolated, genetic drift promotes the random fixation of alleles, thus the number of private alleles among populations can be used as a reference for population connectivity (Hedrick, 2011). We found a high number of private alleles among subspecies (40 in *argyrosperma* and 11 in *sororia*), while the mean number of private alleles per population was similar within subspecies (1.31 in *sororia* and 1.21 in *argyrosperma*).

The number of private alleles found in each subspecies suggests that overall levels of gene flow among subspecies have been low, thus promoting their divergence since the domestication of *argyrosperma* ∼ 8,600 years ago (Rannere et al., 2009). A coalescent based approach such as those implemented in Approximate Bayesian Computation (ABC) analyses, together with a genome-wide approach (thousands of SNPs) will be conducted in the future to test whether these patterns relate to incomplete lineage sorting, ancestral introgression or current introgression.

In *argyrosperma*, we found a high number of private alleles in the populations from the states of Veracruz (Tih) and Oaxaca (Teh). Tih is geographically distant from other sampled populations, and its private alleles may be present in other populations from the Gulf of Mexico; thus, it is advisable to include more populations from this area in further analyses. The population Teh from Oaxaca is located in the area of the Isthmus of Tehuantepec that has been previously identified as an important barrier for the Mexican biota (Ornelas et al., 2013). Moreover, for *sororia*, the population with highest number of private alleles is located in the same area (Soax), further supporting the Isthmus of Tehuantepec as an important biogeographical barrier. Furthermore, seed morphology is distinctive in the populations of *argyrosperma* of Southeastern Mexico, where seeds show clear gray margins in contrast to the golden color found in northern populations (**Figure 1**). People in southeastern Mexico have a strong preference for local varieties (G. Sánchez de la Vega, personal observation). In addition, this may suggest that the high number of private alleles in this area may be related to strong selection pressures associated with seed morphology, as has been reported in *C. pepo* commercial varieties (Formisano et al., 2012). Selection for morphological characters promotes selective sweeps that results in allele fixation in neutral sites of the genome (Meyer and Purugganan, 2013). Alternatively, a high number of private alleles could relate to isolation of these populations. Therefore, we need to conduct genomic and morphologically detailed analyses to test these hypotheses.

The results from the Structure and DAPC analyses show clear genetic differentiation among subspecies (**Figures 3**, **5**). Within *argyrosperma*, Structure analyses (**Figure 3**) show geographically associated groups: (1) a northern group; (2) Yucatan Peninsula; and (3) Pacific coast (**Figure 4**). It is interesting that these genetic groups roughly correspond to the genetic groups reported by Moreno-Estrada et al. (2014) for human Native American populations, which suggest that cultural aspects may be important in determining the genetic structure of this crop. Further analyses should test for the correlations between genetic clusters in domesticated taxa and human Native American populations.

Values of genetic differentiation (*F*_ST_) were variable among populations of both subspecies. *Sororia* showed similar levels of genetic differentiation (*F*_ST_ = 0.243, *R*_ST_ = 0.3) as *argyrosperma* (*F*_ST_ = 0.264, *R*_ST_ = 0.4). In addition, our Mantel test results show that geographically close populations of both subspecies are genetically more similar than expected by chance. Both subspecies have wide distributions (**Figure 2**) that cover a distance of over 1,000 km, thus promoting genetic differentiation among extreme populations. Genetic differentiation in *sororia* could also be related to its patchy distribution and the limited movement (∼0.7 km) and local low densities of its main pollinators from the genera *Peponapis* and *Xenoglossa* (Kohn and Casper, 1992; Montes, 2002; Enríquez et al., 2015). Levels of genetic differentiation among *argyrosperma* populations are similar to those reported for other outcrossing plants (*F*_ST_ = 0.22; Nybom, 2004). It is advisable to include more *sororia* populations in future analyses to determine fine patterns of genetic differentiation.

*F*_ST_ pairwise values are directly related to the degree of phenotypical resemblance among populations and provide insights into their demographic history (Holsinger and Weir, 2009). Our pairwise *F*_ST_ analyses, along all our results of genetic differentiation suggest that both subspecies are genetically well-differentiated.

Also, it is worth noticing that all the analyses of genetic differentiation consistently suggest that the *sororia* populations SoSon and SoSin from the states of Sonora and Sinaloa are more like subspecies *argyrosperma* than *sororia*, including some morphological characteristics of the seeds (**Figure 1**). Our results suggest that these may be escaped populations of *argyrosperma*, as suggested by Merrick and Bates (1989) and Villanueva (2007), who mentioned that individuals from *argyrosperma* are capable of surviving without agricultural management, but that these individuals show a reduction in seed size. Reports suggest that cultivars of *C. pepo* and *C. moschata* in Tamaulipas, Mexico are also capable of surviving and producing fruits in semi-wild or in extreme environmental conditions (Hanselka, 2010).

Gene flow is frequent among subspecies of *Cucurbita* and with other taxa at local levels (Montes-Hernández and Eguiarte, 2002; Lira et al., 2016b). Our analysis suggests that gene flow is less frequent at the regional level, with a few exceptions: (1) in the Yucatan Peninsula, and Chiapas, people mentioned that they usually exchange seeds among neighbors and family members and sell seeds for cultivation, which is consistent with estimated levels of gene flow in this area, and (2) the northern portion of the Pacific Coast, that apparently acts as a genetic corridor that has been previously reported for other crops (Zizumbo-Villarreal and Colunga-GarcíaMarín, 2010).

The results from the barrier analysis are consistent with this idea, where the northern portion of the Sierra Madre Occidental isolates the populations located along the Pacific coast. A pattern of isolation of populations located in Jalisco has also been reported for *Zea mays* ssp. *parviglumis*, the wild relative of maize (Aguirre-Liguori et al., 2017). Finally, when both subspecies were included in the analysis, the Isthmus of Tehuantepec appears as an important barrier, as has been previously reported for many wild taxa (Ornelas et al., 2013).

### Species Distribution Models

Species distribution models of wild relatives of domesticated taxa are a useful tool to corroborate hypotheses of possible domestication sites and environmental suitability for the presence of the wild species (Hufford et al., 2012; Besnard et al., 2013). The SDM for *sororia* suggests that its range has been more or less stable since the mid-Holocene, with possible presence in the Yucatan Peninsula and a more continuous range in Oaxaca and Guerrero during the mid-Holocene (∼6,000 years ago). Many domestication events occurred during this time because of environmental changes and vegetation transitions associated with the end of the Last Glacial Maximum-Holocene, and with the impact of anthropogenic activities (Flannery, 1986; Piperno et al., 2007; Aguirre-Liguori et al., 2016). For the region of Guerrero, Piperno et al. (2007) and Rannere et al. (2009) proposed that the end of the Pleistocene was cold, and as the Holocene advanced this area became warmer, promoting a transition from temperate arboreal elements to tropical forests, environment conditions associated to principal events of domestication in the Balsas basin.

Previous genetic, molecular, biogeographic, and archeological analyses suggest that *argyrosperma* was domesticated in the Balsas-Jalisco region, approximately 9,000 years ago (Sanjur et al., 2002; Piperno et al., 2009; Rannere et al., 2009; Lira et al., 2016b). The oldest archeological remains are from caves located in the Balsas region (Guerrero) from 6,100 to 8,500 years ago. Initial domestication (before 7,000 years ago) was followed by early diversification (Lira et al., 2016b). Our SDM results indicate that environmental characteristics were suitable for the presence of *sororia* in this area during this period.

## Conclusion

Our analyses describe broad patterns of genetic variation, genetic differentiation and gene flow among domesticated and wild *C. argyrosperma*. The levels of genetic variation and genetic differentiation were similar for *sororia* and *argyrosperma*. These could relate to their demographic histories, but further analyses should be conducted to test different demographic hypotheses. Isolation by distance and gene flow analyses suggest that gene flow is more common at a local scale than at a regional scale, perhaps because of pollen movement by specialized pollinators and to human cultural practices, such as seed exchange among populations, but these hypotheses should be tested with ethnobotanical data in future analyses. *Sororia*’s distribution has been relatively stable since the mid-Holocene and suggests the presence of this subspecies in previously described domestication centers based on archeological records. Future analyses should gather information about agricultural management, morphological variation and the behavior of pollinators, along with a wider sampling of the wild populations and the use of massive sequencing data to expand our knowledge of squash domestication.

## Author Contributions

GS-dlV and GC-M contributed to fieldwork, lab work, molecular and population genetics analysis, drafting the manuscript, and final approval of the version to be published. NG contributed to analysis of species distribution models (SDM), drafting the manuscript, and final approval of the version to be published. HH-R contributed to fieldwork, lab work, molecular and data analyses, and final approval of the version to be published. AV-L contributed to laboratory work, logistics, correcting the manuscript, and final approval of the version to be published. EA-P contributed to laboratory work, logistics and molecular analysis, correcting the manuscript, and final approval of the version to be published. JJ-C contributed to correcting the manuscript and final approval of the version to be published. SM-H project leader, contributed to fieldwork, germplasm collection, generated database for project design, and final approval of the version to be published. RL-S project leader, contributed to logistics and final approval of the version to be published. LE project leader, designed and coordinated the project, logistics, drafted and corrected the manuscript, and final approval of the version to be published.

## Conflict of Interest Statement

The authors declare that the research was conducted in the absence of any commercial or financial relationships that could be construed as a potential conflict of interest.

## Footnotes

1 www.biotech.illinois.edu

2 http://beast.bio.ed.ac.uk

3 www.gbif.org
