# Supplementary material for: Genetic Resources in the “Calabaza Pipiana” Squash (Cucurbita argyrosperma) in Mexico: Genetic Diversity, Genetic Differentiation and Distribution Models
Source: Front Plant Sci. 2018 Mar 29;9:400. doi: 10.3389/fpls.2018.00400 (PMC5890117; doi:10.3389/fpls.2018.00400)

Genetic resources in the “calabaza pipiana” squash (*Cucurbita argyrosperma*) in Mexico: Genetic diversity, genetic differentiation and distribution models.

Guillermo Sanchez de la Vega[1], Gabriela Castellanos-Morales[1,2], Niza Gámez[1], Helena S. Hernández-Rosales[1], Alejandra Vázquez-Lobo[1,3], Erika Aguirre-Planter[1], Salvador Montes-Hernández[4], Juan P. Jaramillo-Correa, Rafael Lira-Saade[2*], Luis E. Eguiarte[1*]

Supplementary Table S1. State and population identification of collected samples, number of individuals (n) and decimal coordinates for a) *C. argyrosperma* ssp. *argyrosperma* and b) *C. argyrosperma* ssp. *sororia.* Populations obtained from the germplasm collection of the Instituto Nacional de Investigaciones Forestales, Agrícolas y Pecuarias, Campo Experimental Bajío (BG).

a) *C. argyrosperma* ssp. *argyrosperma*

State	Population	n	Population ID	Coordinates W	Coordinates N
Quintana Roo	Chan_Santa_Cruz	21	Chan	-88.33289167	19.36706389
Yucatán	Ek_Balam	22	Ek	-87.91666667	20.91666667
Yucatán	Motul	22	Mot	-88.82540556	21.24834444
Campeche	Champoton	22	Champ	-90.46137778	19.50115556
Chiapas	Palenque	20	Pal	-91.98776111	17.51281389
Oaxaca	Tehuantepec	20	Teh	-95.23303611	16.33283056
Oaxaca	Mixtepec	21	Mixt	-97.08491667	15.95875
Guerrero	Ometepec	17	Ome	-98.40631944	16.69614722
Guerrero	Matlalapa (BG)	18	Tla	-100.5347222	18.24166667
Guerrero	Tlapehuala (BG)	10	Mtp	-99.59026111	36.36916667
Michoacán	Sahuayo	22	Sah	-102.7162333	20.05871944
Jalisco	Autlán	21	Aut	-104.2043139	19.70195833
Guanajuato	San José Iturbide (BG)	6	SJI	-100.387222	20.998889
San Luis Potosí	Tanquian (BG)	22	Tan	-101.0093306	22.11548611
Veracruz	Tihuatlán (BG)	23	Tih	-97.53950278	20.72006667
Durango	Durango (BG)	6	Dgo	104.583333	24.066667
Coahuila	Cuatrocienegas	22	CCC	-102.066389	26.986111
Sinaloa	Sinaloa (BG)	6	SinalP	-106.0555556	23.27388889
Sonora	Yecora (BG)	6	Yec	-108.9269861	28.37204167

b) *C. argyrosperma* ssp. *sororia*

**State**	**Population**	**n**	**Population ID**	**Coordinates W**	**N**
			Cucurbita argyrosperma ssp. sororia		
Chiapas	Chiapas	20	20. SChis	-93.626878	16.597486
Oaxaca	Oaxaca	21	21.SOax	-97.076308	15.918225
Guerrero	Guerrero	22	22. SGro	-98.406319	16.688139
Jalisco	Jalisco	22	23.SJal	-104.2043139	19.70195833
Sinaloa	Sinaloa (BG)	13	24.SoSin	-106.416667	24
Sonora	Sonora (BG)	15	25.SoSon	-109.583333	28.533333

Supplementary Table S2. List of nuclear microsatellite loci (Gong et al., 2008) used in the current analysis of *Cucurbita argyrosperma*.

Marker name	Alleles	Motif	No.of Repeats	Forward primer	Reverse primer	Expected size (bp)	Annealing temperature (°C)
CMTp17	4	CT	16	ACTGCTCAATAAGGCAAGGA	AAACAAGAGTGCACAAACAGG	84	58
CMTp88	5	TC	12	ACCTACCGTCACACCCACAT	CCACCTGAAAACAGGGCTAA	167	60
CMTp129	5	AG	19	CTCTTGCTCATCTTCCTTGTTG	CCCACCCATTTACCCTCTAGT	147	59
CMTp175	5	GA	11	TCCAATGCACAACCTTGC	GCCTCGGTTTTTGTCAAGAT	155	59
CMTp193	5	GA	18	GGTGACGGCAAGAAAAGCTA	GCTGACCCTCTCTCCCTCTC	186	60
CMTm11	5	AG	14	TGGAAGGATTCTCCCACAGT	TACAATTTGACGTCCGCAAG	108	59
CMTm54	5	CT	15	GTGTGGATGCAAATGGTGAG	GGGAATCGAGGGTTTTGAAT	143	60
CMTm120	4	CT	13	GCCAAAGGTTCCAAATGACA	TGATTTGCGCACAaACAAAC	121	60
CMTm144	4	AG	11	ACATGGGCATACCTCGAATC	CACCTGGCTGTTTTGTCTGA	150	60
CMTm187	4	AG	19	ATCGGTGAGTCCCAAAAATG	ATCACAAAGCGGGAAAACAC	128	60
CMTm221	4	CT	11	CAATAAGATAGCTCTCACGTTGC	TGCCTAGTTATCGCGACTTC	108	58
CMTm261	4	A	21	GGTGGCCTCTGAACAATTTC	ACCTAACCAATGGGCATGAG	228	60

Supplementary Table S3. *Cucurbita argyrosperma* populations sampled and loci with significant departures from Hardy–Weinberg (HW) equilibrium (* $p < 0.05$ and ** $p < 0.001$). Values correspond to F_{IS} estimates for each locus and each population. Population ID from Supplementary Table S1.

Population ID	CMTp17	CMTm120	CMTm261	CMTm11	CMTp129	CMTp193	CMTm221	CMTm54	CMTp88	Multi-locus
Chan	0.068	-0.2	---	---	0.647**	---	---	---	0.786**	0.374**
Ek	-0.629**	-0.235	---	-0.105	-0.105	-0.235	---	0	1*	-0.254*
Mot	0.05	0.35	---	-0.077	-0.286	0	-0.053	-0.11	0.661*	0.052
Champ	-0.105	0.045	---	---	0.469**	-0.135	-0.05	0	1**	0.277**
Pal	-0.67**	-0.103	0	-0.132	0.13	-0.166	---	-0.187	-0.027	-0.189*
Teh	-0.299	-0.09	-0.056	1*	0.013	0.13	0.159	0.172	---	0.038
Mix	-0.316	0.588*	---	-0.053	-0.101	-0.212	0.355*	1*	---	0.12*
Ome	-0.387	-0.439	---	-0.867*	0.222	-0.481	0.65	-0.077	-0.12	-0.26
Tla	0.196	0.273	---	-0.346	0.386	-0.286	---	0	---	0.018
Mtp	-0.172	0.15	---	-0.259	-0.684*	-0.417	-0.083	0	0.382	-0.174*
Sah	-0.11	0.129	---	-0.312	-0.503*	-0.448	-0.024	---	---	-0.26**
Aut	0.096	0.355	---	-0.176	0.395**	-0.073	---	---	0	0.131*
SJI	-0.091	-0.25	0.333	-0.667	---	---	-0.429	---	1	-0.067
Tan	0.173	0.332*	1**	-0.135	-0.077	-0.312	---	-0.14	---	0.203*
Tih	0.509*	0.333	---	-0.193	0.134	-0.294	0.355*	0.662*	1**	0.238**
Dgo	1*	-0.25	---	-0.25	0	-0.111	0.062	-0.429	-0.25	0.04*
CCC	-0.279	-0.188	0.894**	1*	0	-0.293	---	-0.07	0.878**	0.128**
SinalP	0.5	---	-1	---	---	-0.333	---	-0.333	1	0.059
Yec	-0.667	0.259	---	0.167	0.4	1*	---	-0.333	0	0.185*
SChis	0.823*	0.237	0.202	---	---	-0.691*	---	-0.267	---	0.085
SOax	0.2	-0.135	---	---	1**	-0.6	---	0.707**	---	0.304**
SGro	1	0.36*	---	-0.024	0.131	-0.294	-0.05	1**	---	0.225*
SJal	1*	0.148	---	-0.448	0.601**	-0.273	---	-0.333*	---	0.035
SoSin	---	-0.352*	-0.037	-0.251	0.783**	-0.333	-0.043	-0.143	---	-0.107
SoSon	---	0.248	-0.077	0.012	-0.077	-0.125	---	---	---	-0.013
Overall	0.017	0.068	0.148*	-0.235*	0.271**	-0.226**	0.06**	-0.019	0.599**	0.043**

Supplementary Table S4. Matrix of estimates of recent migration rates among populations and subspecies of *Cucurbita argyrosperma* obtained with BayesAss based on 12 nuclear microsatellite loci. The proportion of non-migrants is shown in the main diagonal (in bold). The migration matrix is presented in three parts: a) populations 1 – 10 of *C. argyrosperma* ssp. *argyrosperma*; b) populations 11 – 19 of *C. argyrosperma* ssp. *argyrosperma*; and c) populations 20 – 25 correspond to *C. argyrosperma* ssp. *sororia* (shaded in light gray). Population ID from Supplementary Table S1.

a) Populations 1 – 10 of *C. argyrosperma* ssp. *argyrosperma*

Population ID	1. Chan	2. Ek	3. Mot	4. Champ	5.Pal	6. Teh	7. Mixt	8.Ome	9.Tla	10.Mtp
**1. Chan**	**0.8175 (0.0251)**	0.0159 (0.0135)	0.0070 (0.0069)	0.0073 (0.0070)	0.0069 (0.0068)	0.0069 (0.0070)	0.0071 (0.0070)	0.0071 (0.0070)	0.0070 (0.0069)	0.0075 (0.0076)
**2. Ek**	0.0087 (0.0086)	**0.8260 (0.0249)**	0.0069 (0.0068)	0.0072 (0.0072)	0.0072 (0.0070)	0.0071 (0.0072)	0.0072 (0.0072)	0.0068 (0.0068)	0.0072 (0.0068)	0.0068 (0.0068)
**3. Mot**	0.0101 (0.0094)	0.1527 (0.0233)	**0.6740 (0.0072)**	0.0071 (0.0070)	0.0072 (0.0070)	0.0071 (0.0070)	0.0074 (0.0072)	0.0071 (0.0070)	0.0073 (0.0071)	0.0073 (0.0072)
**4. Champ**	0.1385 (0.0259)	0.0288 (0.0166)	0.0072 (0.0070)	**0.6739 (0.0071)**	0.0072 (0.0071)	0.0072 (0.0071)	0.0072 (0.0071)	0.0071 (0.0070)	0.0071 (0.0069)	0.0074 (0.0072)
**5.Pal**	0.0095 (0.0090)	0.1548 (0.0252)	0.0073 (0.0072)	0.0073 (0.0072)	**0.6741 (0.0072)**	0.0073 (0.0072)	0.0073 (0.0071)	0.0074 (0.0072)	0.0075 (0.0073)	0.0073 (0.0070)
**6. Teh**	0.0222 (0.0147)	0.0140 (0.0124)	0.0073 (0.0072)	0.0076 (0.0075)	0.0074 (0.0074)	**0.7841 (0.0265)**	0.0095 (0.0093)	0.0076 (0.0073)	0.0075 (0.0074)	0.0150 (0.0130)
**7. Mixt**	0.0074 (0.0072)	0.0082 (0.0076)	0.0069 (0.0065)	0.0071 (0.0069)	0.0074 (0.0071)	0.0088 (0.0086)	**0.8135 (0.0251)**	0.0072 (0.0072)	0.0075 (0.0073)	0.0075 (0.0072)
**8.Ome**	0.0084 (0.0081)	0.0083 (0.0080)	0.0082 (0.0081)	0.0083 (0.0083)	0.0085 (0.0083)	0.0082 (0.0080)	0.0087 (0.0085)	**0.6750 (0.0081)**	0.0083 (0.0081)	0.0083 (0.0083)
**9.Tla**	0.0160 (0.0138)	0.0221 (0.0181)	0.0095 (0.0091)	0.0096 (0.0095)	0.0096 (0.0093)	0.0095 (0.0091)	0.0116 (0.0109)	0.0095 (0.0092)	**0.6762 (0.0093)**	0.0095 (0.0092)
**10.Mtp**	0.0079 (0.0077)	0.0093 (0.0088)	0.0080 (0.0078)	0.0079 (0.0075)	0.0079 (0.0077)	0.0096 (0.0095)	0.0082 (0.0077)	0.0076 (0.0074)	0.0075 (0.0077)	**0.8002 (0.0263)**
**11.Sah**	0.0158 (0.0110)	0.1477 (0.0243)	0.0073 (0.0071)	0.0072 (0.0071)	0.0071 (0.0071)	0.0073 (0.0071)	0.0076 (0.0074)	0.0072 (0.0069)	0.0069 (0.0069)	0.0070 (0.0068)
**12.Aut**	0.0073 (0.0074)	0.0090 (0.0087)	0.0072 (0.0069)	0.0073 (0.0072)	0.0073 (0.0069)	0.0083 (0.0084)	0.0092 (0.0088)	0.0073 (0.0071)	0.0071 (0.0073)	0.0079 (0.0078)
**13.SJI**	0.0106 (0.0102)	0.0108 (0.0104)	0.0106 (0.0102)	0.0109 (0.0105)	0.0109 (0.0105)	0.0105 (0.0102)	0.0758 (0.0248)	0.0107 (0.0104)	0.0108 (0.0104)	0.0108 (0.0104)

Population ID	1. Chan	2. Ek	3. Mot	4. Champ	5.Pal	6. Teh	7. Mixt	8.Ome	9.Tla	10.Mtp
**14.Tan**	0.0073<br>(0.0072)	0.0092<br>(0.0090)	0.0069<br>(0.0069)	0.0072<br>(0.0070)	0.0071<br>(0.0071)	0.0070<br>(0.0068)	0.0691<br>(0.0234)	0.0073<br>(0.0071)	0.0070<br>(0.0067)	0.0072<br>(0.0072)
**15.Tih**	0.0069<br>(0.0067)	0.0072<br>(0.0068)	0.0072<br>(0.0067)	0.0070<br>(0.0068)	0.0069<br>(0.0065)	0.0072<br>(0.0070)	0.0073<br>(0.0074)	0.0070<br>(0.0070)	0.0072<br>(0.0070)	0.0072<br>(0.0068)
**16.Dgo**	0.0108<br>(0.0106)	0.0108<br>(0.0104)	0.0107<br>(0.0106)	0.0107<br>(0.0104)	0.0109<br>(0.0105)	0.0108<br>(0.0105)	0.0109<br>(0.0107)	0.0107<br>(0.0104)	0.0108<br>(0.0104)	0.0110<br>(0.0107)
**17.CCC**	0.0076<br>(0.0074)	0.0070<br>(0.0070)	0.0072<br>(0.0070)	0.0073<br>(0.0073)	0.0073<br>(0.0073)	0.0071<br>(0.0068)	0.0072<br>(0.0072)	0.0076<br>(0.0073)	0.0074<br>(0.0070)	0.0073<br>(0.0072)
**18.SinalP**	0.0119<br>(0.0115)	0.0119<br>(0.0116)	0.0119<br>(0.0116)	0.0121<br>(0.0116)	0.0118<br>(0.0115)	0.0118<br>(0.0113)	0.0119<br>(0.0116)	0.0119<br>(0.0115)	0.0118<br>(0.0114)	0.0122<br>(0.0116)
**19. Yec**	0.0107<br>(0.0103)	0.0109<br>(0.0105)	0.0105<br>(0.0103)	0.0106<br>(0.0104)	0.0106<br>(0.0103)	0.0107<br>(0.0103)	0.0113<br>(0.0107)	0.0106<br>(0.0104)	0.0106<br>(0.0102)	0.0107<br>(0.0105)
**20. SChis**	0.0073<br>(0.0072)	0.0073<br>(0.0073)	0.0071<br>(0.0069)	0.0073<br>(0.0072)	0.0074<br>(0.0072)	0.0074<br>(0.0074)	0.0075<br>(0.0072)	0.0078<br>(0.0079)	0.0075<br>(0.0076)	0.0073<br>(0.0072)
**21.SOax**	0.0073<br>(0.0071)	0.0072<br>(0.0069)	0.0072<br>(0.0072)	0.0073<br>(0.0069)	0.0073<br>(0.0070)	0.0074<br>(0.0072)	0.0076<br>(0.0077)	0.0076<br>(0.0073)	0.0072<br>(0.0072)	0.0074<br>(0.0074)
**22. SGro**	0.0072<br>(0.0069)	0.0071<br>(0.0070)	0.0069<br>(0.0067)	0.0068<br>(0.0068)	0.0074<br>(0.0069)	0.0070<br>(0.0068)	0.0071<br>(0.0067)	0.0072<br>(0.0071)	0.0070<br>(0.0071)	0.0072<br>(0.0070)
**23.SJal**	0.0073<br>(0.0071)	0.0070<br>(0.0068)	0.0068<br>(0.0066)	0.0070<br>(0.0069)	0.0074<br>(0.0073)	0.0070<br>(0.0066)	0.0071<br>(0.0068)	0.0072<br>(0.0069)	0.0072<br>(0.0069)	0.0069<br>(0.0068)
**24.SoSin**	0.0088<br>(0.0086)	0.0088<br>(0.0085)	0.0085<br>(0.0083)	0.0089<br>(0.0086)	0.0088<br>(0.0085)	0.0088<br>(0.0085)	0.0087<br>(0.0085)	0.0088<br>(0.0086)	0.0088<br>(0.0085)	0.0093<br>(0.0093)
**25.SoSon**	0.0084<br>(0.0083)	0.0085<br>(0.0084)	0.0084<br>(0.0082)	0.0083<br>(0.0080)	0.0082<br>(0.0081)	0.0083<br>(0.0082)	0.0084<br>(0.0081)	0.0084<br>(0.0081)	0.0085<br>(0.0082)	0.0083<br>(0.0081)

b) Populations 11 – 19 of *C. argyrosperma* ssp. *argyrosperma*

Population ID	11.Sah	12.Aut	13.SJI	14.Tan	15.Tih	16.Dgo	17.CCC	18.SinalP	19. Yec
**1. Chan**	0.0080 (0.0076)	0.0072 (0.0070)	0.0070 (0.0069)	0.0091 (0.0083)	0.0072 (0.0073)	0.0073 (0.0072)	0.0075 (0.0075)	0.0074 (0.0071)	0.0069 (0.0067)
**2. Ek**	0.0089 (0.0084)	0.0070 (0.0070)	0.0072 (0.0069)	0.0076 (0.0077)	0.0070 (0.0068)	0.0070 (0.0071)	0.0070 (0.0072)	0.0072 (0.0070)	0.0071 (0.0069)
**3. Mot**	0.0072 (0.0070)	0.0072 (0.0071)	0.0071 (0.0069)	0.0119 (0.0103)	0.0073 (0.0070)	0.0072 (0.0071)	0.0073 (0.0072)	0.0072 (0.0072)	0.0072 (0.0071)
**4. Champ**	0.0088 (0.0084)	0.0072 (0.0070)	0.0070 (0.0068)	0.0070 (0.0068)	0.0072 (0.0071)	0.0071 (0.0070)	0.0071 (0.0070)	0.0072 (0.0069)	0.0071 (0.0070)
**5.Pal**	0.0076 (0.0075)	0.0074 (0.0072)	0.0074 (0.0073)	0.0074 (0.0073)	0.0075 (0.0073)	0.0074 (0.0071)	0.0072 (0.0070)	0.0074 (0.0072)	0.0072 (0.0071)
**6. Teh**	0.0092 (0.0090)	0.0117 (0.0099)	0.0076 (0.0071)	0.0082 (0.0077)	0.0073 (0.0072)	0.0073 (0.0070)	0.0075 (0.0075)	0.0074 (0.0073)	0.0073 (0.0071)
**7. Mixt**	0.0097 (0.0093)	0.0138 (0.0115)	0.0074 (0.0071)	0.0071 (0.0072)	0.0074 (0.0071)	0.0076 (0.0072)	0.0074 (0.0071)	0.0064 (0.0065)	0.0074 (0.0073)
**8.Ome**	0.1329 (0.0254)	0.0083 (0.0082)	0.0084 (0.0082)	0.0084 (0.0082)	0.0083 (0.0081)	0.0084 (0.0081)	0.0084 (0.0083)	0.0084 (0.0082)	0.0083 (0.0081)
**9.Tla**	0.0450 (0.0222)	0.0342 (0.0201)	0.0095 (0.0093)	0.0232 (0.0160)	0.0095 (0.0093)	0.0097 (0.0092)	0.0097 (0.0092)	0.0096 (0.0092)	0.0095 (0.0093)
**10.Mtp**	0.0083 (0.0079)	0.0115 (0.0112)	0.0077 (0.0076)	0.0125 (0.0102)	0.0080 (0.0079)	0.0078 (0.0074)	0.0077 (0.0076)	0.0077 (0.0076)	0.0077 (0.0075)
**11.Sah**	**0.6762 (0.0092)**	0.0081 (0.0080)	0.0072 (0.0070)	0.0081 (0.0077)	0.0076 (0.0074)	0.0072 (0.0070)	0.0072 (0.0070)	0.0071 (0.0069)	0.0072 (0.0071)
**12.Aut**	0.0088 (0.0087)	**0.8174 (0.0252)**	0.0073 (0.0074)	0.0090 (0.0086)	0.0076 (0.0076)	0.0068 (0.0066)	0.0073 (0.0073)	0.0073 (0.0071)	0.0074 (0.0073)
**13.SJI**	0.0106 (0.0102)	0.0107 (0.0103)	**0.6774 (0.0104)**	0.0106 (0.0103)	0.0109 (0.0104)	0.0108 (0.0105)	0.0107 (0.0103)	0.0104 (0.0101)	0.0108 (0.0106)
**14.Tan**	0.0075 (0.0072)	0.0111 (0.0107)	0.0070 (0.0067)	**0.7606 (0.0249)**	0.0074 (0.0070)	0.0072 (0.0070)	0.0071 (0.0071)	0.0071 (0.0069)	0.0071 (0.0068)
**15.Tih**	0.0071 (0.0069)	0.0128 (0.0095)	0.0071 (0.0071)	0.0069 (0.0066)	**0.8262 (0.0237)**	0.0067 (0.0065)	0.0068 (0.0066)	0.0068 (0.0067)	0.0070 (0.0071)
**16.Dgo**	0.0109 (0.0105)	0.0111 (0.0105)	0.0109 (0.0104)	0.0110 (0.0107)	0.0109 (0.0105)	**0.6776 (0.0106)**	0.0735 (0.0251)	0.0107 (0.0104)	0.0107 (0.0102)

Population ID	11.Sah	12.Aut	13.SJI	14.Tan	15.Tih	16.Dgo	17.CCC	18.SinalP	19. Yec
**17.CCC**	0.0072 (0.0069)	0.0072 (0.0071)	0.0074 (0.0073)	0.0073 (0.0071)	0.0075 (0.0073)	0.0071 (0.0072)	**0.8253 (0.0245)**	0.0072 (0.0070)	0.0072 (0.0071)
**18.SinalP**	0.0118 (0.0113)	0.0119 (0.0114)	0.0120 (0.0117)	0.0337 (0.0195)	0.0130 (0.0127)	0.0119 (0.0116)	0.0248 (0.0171)	**0.6785 (0.0115)**	0.0119 (0.0115)
**19. Yec**	0.0107 (0.0105)	0.0766 (0.0253)	0.0106 (0.0103)	0.0107 (0.0104)	0.0105 (0.0104)	0.0106 (0.0103)	0.0106 (0.0104)	0.0107 (0.0101)	**0.6774 (0.0105)**
**20. SChis**	0.0073 (0.0071)	0.0073 (0.0070)	0.0076 (0.0072)	0.0079 (0.0076)	0.0076 (0.0073)	0.0073 (0.0072)	0.0078 (0.0076)	0.0073 (0.0070)	0.0076 (0.0071)
**21.SOax**	0.0074 (0.0069)	0.0071 (0.0070)	0.0074 (0.0070)	0.0069 (0.0068)	0.0074 (0.0072)	0.0070 (0.0068)	0.0068 (0.0067)	0.0072 (0.0070)	0.0071 (0.0070)
**22. SGro**	0.0070 (0.0069)	0.0072 (0.0069)	0.0073 (0.0070)	0.0069 (0.0066)	0.0072 (0.0071)	0.0072 (0.0071)	0.0070 (0.0069)	0.0073 (0.0071)	0.0072 (0.0068)
**23.SJal**	0.0067 (0.0067)	0.0070 (0.0070)	0.0072 (0.0073)	0.0068 (0.0068)	0.0073 (0.0070)	0.0073 (0.0073)	0.0074 (0.0071)	0.0071 (0.0070)	0.0074 (0.0072)
**24.SoSin**	0.0086 (0.0085)	0.0099 (0.0097)	0.0088 (0.0085)	0.1221 (0.0261)	0.0088 (0.0084)	0.0086 (0.0084)	0.0086 (0.0085)	0.0088 (0.0085)	0.0087 (0.0085)
**25.SoSon**	0.0085 (0.0082)	0.0083 (0.0081)	0.0083 (0.0080)	0.1324 (0.0247)	0.0084 (0.0080)	0.0083 (0.0081)	0.0082 (0.0079)	0.0085 (0.0082)	0.0085 (0.0082)

c) Populations 20 – 25 correspond to *C. argyrosperma* ssp. *sororia*

Population ID	20. SChis	21.SOax	22. SGro	23.SJal	24.SoSin	25.SoSon
**1. Chan**	0.0068 (0.0067)	0.0071 (0.0069)	0.0071 (0.0068)	0.0074 (0.0069)	0.0068 (0.0069)	0.0070 (0.0070)
**2. Ek**	0.0072 (0.0068)	0.0076 (0.0074)	0.0072 (0.0070)	0.0072 (0.0071)	0.0072 (0.0071)	0.0069 (0.0067)
**3. Mot**	0.0072 (0.0070)	0.0072 (0.0071)	0.0073 (0.0071)	0.0073 (0.0071)	0.0071 (0.0069)	0.0071 (0.0069)
**4. Champ**	0.0072 (0.0070)	0.0072 (0.0068)	0.0070 (0.0068)	0.0071 (0.0068)	0.0071 (0.0069)	0.0070 (0.0069)
**5.Pal**	0.0074 (0.0072)	0.0073 (0.0072)	0.0072 (0.0072)	0.0072 (0.0070)	0.0073 (0.0070)	0.0074 (0.0072)
**6. Teh**	0.0074 (0.0071)	0.0075 (0.0075)	0.0071 (0.0072)	0.0075 (0.0072)	0.0073 (0.0072)	0.0075 (0.0072)

Population ID	20. SChis	21.SOax	22. SGro	23.SJal	24.SoSin	25.SoSon
**7. Mixt**	0.0075 (0.0071)	0.0075 (0.0070)	0.0073 (0.0071)	0.0074 (0.0073)	0.0073 (0.0072)	0.0073 (0.0073)
**8.Ome**	0.0084 (0.0082)	0.0083 (0.0081)	0.0084 (0.0082)	0.0084 (0.0082)	0.0082 (0.0082)	0.0082 (0.0080)
**9.Tla**	0.0095 (0.0092)	0.0095 (0.0092)	0.0095 (0.0093)	0.0097 (0.0093)	0.0096 (0.0094)	0.0095 (0.0092)
**10.Mtp**	0.0079 (0.0078)	0.0078 (0.0075)	0.0079 (0.0078)	0.0078 (0.0076)	0.0078 (0.0074)	0.0078 (0.0078)
**11.Sah**	0.0071 (0.0070)	0.0072 (0.0069)	0.0072 (0.0071)	0.0072 (0.0069)	0.0072 (0.0071)	0.0072 (0.0069)
**12.Aut**	0.0075 (0.0071)	0.0068 (0.0067)	0.0075 (0.0075)	0.0070 (0.0068)	0.0072 (0.0072)	0.0072 (0.0072)
**13.SJI**	0.0106 (0.0102)	0.0108 (0.0106)	0.0109 (0.0107)	0.0109 (0.0104)	0.0106 (0.0103)	0.0109 (0.0106)
**14.Tan**	0.0072 (0.0069)	0.0071 (0.0069)	0.0071 (0.0069)	0.0072 (0.0069)	0.0072 (0.0070)	0.0070 (0.0069)
**15.Tih**	0.0068 (0.0067)	0.0072 (0.0069)	0.0067 (0.0064)	0.0069 (0.0068)	0.0069 (0.0068)	0.0070 (0.0064)
**16.Dgo**	0.0108 (0.0104)	0.0108 (0.0103)	0.0108 (0.0104)	0.0109 (0.0106)	0.0108 (0.0104)	0.0108 (0.0105)
**17.CCC**	0.0073 (0.0071)	0.0073 (0.0070)	0.0078 (0.0075)	0.0071 (0.0072)	0.0073 (0.0070)	0.0069 (0.0068)
**18.SinalP**	0.0117 (0.0114)	0.0121 (0.0115)	0.0116 (0.0113)	0.0120 (0.0116)	0.0120 (0.0117)	0.0120 (0.0115)
**19. Yec**	0.0108 (0.0105)	0.0108 (0.0106)	0.0106 (0.0104)	0.0109 (0.0106)	0.0106 (0.0104)	0.0107 (0.0103)
**20. SChis**	**0.8219 (0.0246)**	0.0074 (0.0073)	0.0074 (0.0070)	0.0076 (0.0075)	0.0073 (0.0071)	0.0070 (0.0068)
**21.SOax**	0.0069 (0.0072)	**0.8138 (0.0245)**	0.0198 (0.0120)	0.0073 (0.0070)	0.0070 (0.0070)	0.0073 (0.0073)
**22. SGro**	0.0071 (0.0069)	0.0084 (0.0081)	**0.8280 (0.0238)**	0.0073 (0.0069)	0.0071 (0.0072)	0.0070 (0.0068)
**23.SJal**	0.0076 (0.0072)	0.0073 (0.0073)	0.0202 (0.0122)	**0.8163 (0.0241)**	0.0068 (0.0070)	0.0068 (0.0066)

Population ID	20. SChis	21.SOax	22. SGro	23.SJal	24.SoSin	25.SoSon
**24.SoSin**	0.0087 (0.0084)	0.0088 (0.0087)	0.0087 (0.0084)	0.0087 (0.0084)	**0.6753 (0.0084)**	0.0088 (0.0085)
**25.SoSon**	0.0085 (0.0082)	0.0082 (0.0081)	0.0082 (0.0081)	0.0086 (0.0083)	0.0084 (0.0082)	**0.6751 (0.0083)**

Supplementary Table S5. Matrix of genetic differentiation among populations (pairwise F_{ST}) among populations and subspecies of *Cucurbita argyrosperma* obtained with Arlequin based on 12 nuclear microsatellite loci. The pairwise F_{ST} matrix is presented in three parts: a) populations 1 – 10 of *C. argyrosperma* ssp. *argyrosperma*; b) populations 11 – 19 of *C. argyrosperma* ssp. *argyrosperma*; and c) populations 20 – 25 correspond to *C. argyrosperma* ssp. *sororia* (shaded in light gray). Population ID from Supplementary Table S1.

a) Populations 1 – 10 of *C. argyrosperma* ssp. *Argyrosperma*

Population ID	1. Chan	2. Ek	3. Mot	4. Champ	5.Pal	6. Teh	7. Mixt	8.Ome	9.Tla	10.Mtp
**1. Chan**	0									
**2. Ek**	0.02741	0								
**3. Mot**	0.0592	0.03482	0							
**4. Champ**	0.02577	0.0494	0.08443	0						
**5.Pal**	0.0926	0.03331	0.00885	0.08776	0					
**6. Teh**	0.17231	0.1687	0.10328	0.14972	0.06534	0				
**7. Mixt**	0.05978	0.0012	0.04653	0.08369	0.0341	0.13485	0			
**8.Ome**	0.08978	0.05533	0.06342	0.04811	0.00681	0.05657	0.07044	0		
**9.Tla**	0.35304	0.25026	0.31241	0.30027	0.16971	0.22946	0.27528	0.10875	0	
**10.Mtp**	0.09725	0.03096	0.09175	0.14515	0.05503	0.15308	0.0207	0.07152	0.22706	0
**11.Sah**	0.14006	0.17401	0.24273	0.08925	0.17739	0.18588	0.20095	0.07981	0.27858	0.21564
**12.Aut**	0.28378	0.16019	0.2522	0.25804	0.12347	0.22838	0.15126	0.12199	0.07888	0.14556
**13.SJI**	0.16032	0.03345	0.05536	0.21116	0.04814	0.14047	0.00814	0.14816	0.36232	0.0852
**14.Tan**	0.1242	0.03497	0.04902	0.13945	0.0075	0.12757	0.01113	0.06928	0.27529	0.07116

**15.Tih**	0.82554	0.78641	0.76164	0.83157	0.71457	0.73646	0.79548	0.76551	0.77942	0.78992
**16.Dgo**	0.30611	0.16352	0.11862	0.34433	0.02655	0.10667	0.16113	0.1507	0.31989	0.16051
**17.CCC**	0.35035	0.21154	0.13722	0.31787	0.03218	0.13116	0.24259	0.12782	0.27555	0.2543
**18.SinalP**	0.30243	0.09777	0.14611	0.27624	0.01099	0.14903	0.12108	0.05725	0.05024	0.10219
**19. Yec**	0.38352	0.34345	0.29131	0.37018	0.16951	0.0749	0.32098	0.147	0.17083	0.30615
**20. SChis**	0.85536	0.83268	0.77236	0.84621	0.6997	0.61162	0.84615	0.72858	0.80024	0.84191
**21.SOax**	0.79956	0.78146	0.72838	0.78967	0.66561	0.58479	0.78649	0.67619	0.72777	0.7793
**22. SGro**	0.22541	0.13578	0.07133	0.21882	0.02522	0.12885	0.14881	0.07901	0.26653	0.13635
**23.SJal**	0.66459	0.6214	0.54596	0.65173	0.45466	0.39877	0.62101	0.49116	0.54373	0.61042
**24.SoSin**	0.37773	0.21982	0.27705	0.34866	0.14276	0.28051	0.26515	0.15354	0.07521	0.2123
**25.SoSon**	0.23619	0.06986	0.14734	0.2423	0.05967	0.22991	0.08849	0.11763	0.2109	0.08823

b) Populations 11 – 19 of *C. argyrosperma* ssp. *Argyrosperma*

Population ID	11.Sah	12.Aut	13.SJI	14.Tan	15.Tih	16.Dgo	17.CCC	18.SinalP	19. Yec
**11.Sah**	0								
**12.Aut**	0.25626	0							
**13.SJI**	0.42804	0.28132	0						
**14.Tan**	0.25444	0.13708	0.11845	0					
**15.Tih**	0.86769	0.7898	0.74086	0.79732	0				
**16.Dgo**	0.46416	0.27599	0.22785	0.18008	0.59151	0			
**17.CCC**	0.45107	0.30276	0.33911	0.21569	0.68731	0.23085	0		
**18.SinalP**	0.42557	0.02366	0.57814	0.10579	0.7563	0.25044	0.14034	0	
**19. Yec**	0.35721	0.26056	0.28004	0.28458	0.74306	0.14127	0.27448	0.15186	0
**20. SChis**	0.8793	0.84727	0.90702	0.84225	0.90218	0.83282	0.7193	0.88424	0.6082
**21.SOax**	0.8149	0.78817	0.78989	0.77844	0.86421	0.71462	0.67158	0.74916	0.5002
**22. SGro**	0.3515	0.26178	0.1763	0.13839	0.73825	0.1465	0.06933	0.09438	0.27937
**23.SJal**	0.69549	0.61771	0.60556	0.59612	0.75152	0.47444	0.37922	0.51322	0.3159
**24.SoSin**	0.43157	0.13943	0.4114	0.25751	0.77505	0.36876	0.22962	-0.04492	0.32879
**25.SoSon**	0.36797	0.09552	0.23904	0.0776	0.78815	0.2746	0.25734	-0.03218	0.33728

c) Populations 20 – 25 correspond to *C. argyrosperma* ssp. *sororia*

**POPULATION**	**20. SChis**	**21.SOax**	**22. SGro**	**23.SJal**	**24.SoSin**	**25.SoSon**
**1. Chan**						
**2. Ek**						
**3. Mot**						
**4. Champ**						
**5.Pal**						
**6. Teh**						
**7. Mixt**						
**8.Ome**						
**9.Tla**						
**10.Mtp**						
**11.Sah**						
**12.Aut**						
**13.SJI**						
**14.Tan**						
**15.Tih**						
**16.Dgo**						
**17.CCC**						
**18.SinalP**						
**19. Yec**						
**20. SChis**	0					
**21.SOax**	0.06682	0				
**22. SGro**	0.72271	0.68373	0			
**23.SJal**	0.21812	0.25969	0.4521	0		
**24.SoSin**	0.85007	0.77283	0.19354	0.57232	0	
**25.SoSon**	0.87895	0.79829	0.15187	0.61453	0.57232	0

Supplementary Figure S1. Distribution of 273 occurrence points of *C. argyrosperma* ssp. *sororia* used for the species distribution model (SDM).

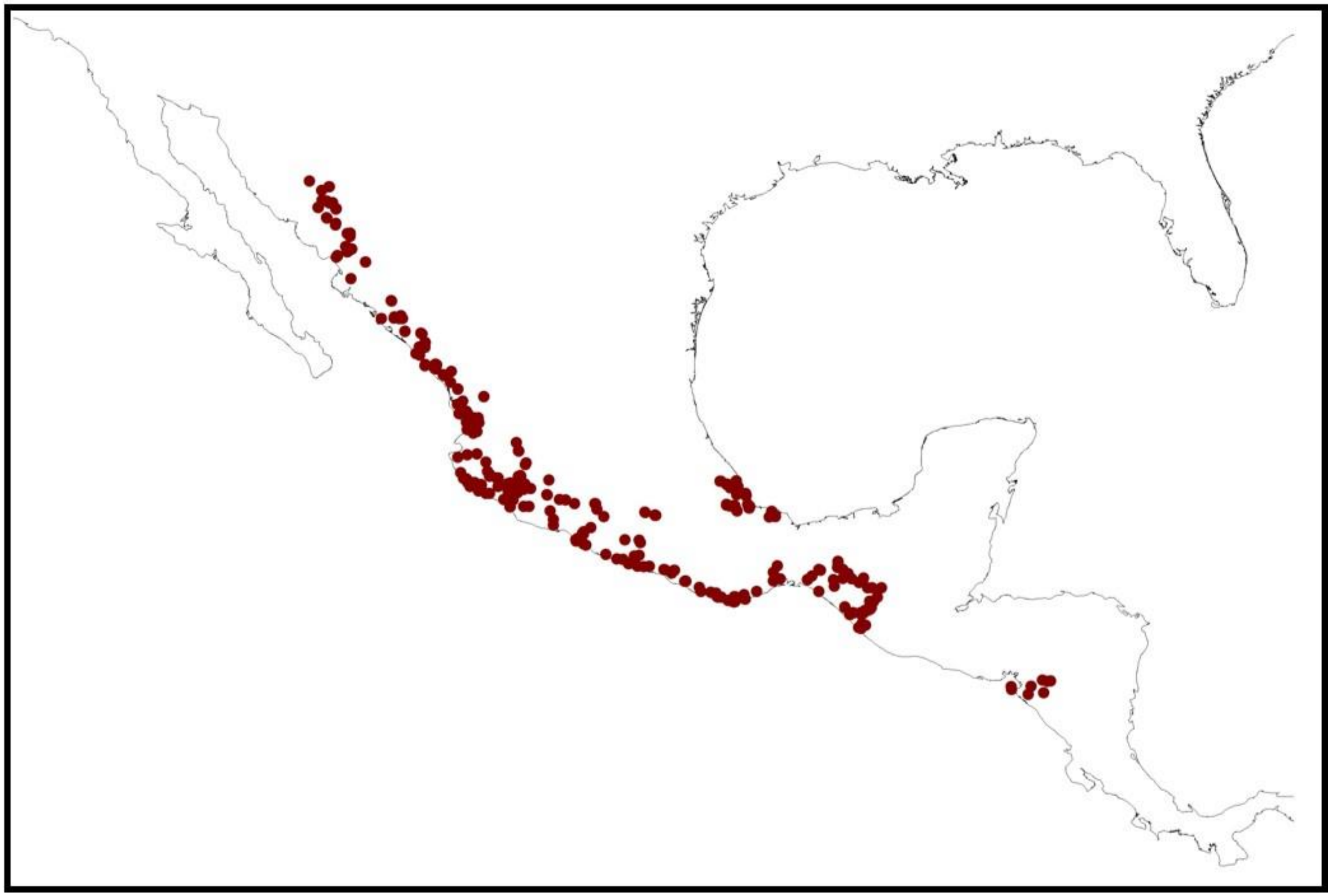

Supplement: Supplementary file 1 [file Data_Sheet_1.PDF]
